# Hybridizing deep learning algorithms and geostatistical approaches for improved crop yield disaggregation

**DOI:** 10.1371/journal.pone.0344081

**Published:** 2026-03-06

**Authors:** Saravanakumar R., Rajni Jain, Vaibhav Kumar Singh, Anshu Bharadwaj, Vinay Kumar Sehgal, Ankur Biswas, Alka Arora, Hari Krishna

**Affiliations:** 1 ICAR- Indian Agricultural Research Institute, New Delhi, India; 2 ICAR- Indian Agricultural Statistical Research Institute, New Delhi, India; 3 ICAR- National Institute of Agricultural Economics and Policy Research, New Delhi, India; Swedish Meteorological and Hydrological Institute, SWEDEN

## Abstract

Reliable crop yield estimates at fine spatial resolution are essential for precision agriculture, food security planning, and insurance schemes. However, yield statistics are reported at coarser administrative levels, limiting their applicability for field-scale analysis. This study proposes a multi-stage hybridized framework that integrates deep learning (DL) models with geostatistical residual kriging to disaggregate village-level crop yield statistics to the pixel level. The proposed methodology is demonstrated using wheat and mustard crops as case study in the semi-arid districts, Haryana, India. The study identifies suitable data combination by evaluating multiple combinations of soil, weather, Sentinel-1, and Sentinel-2 bands data for yield disaggregation. Results show that datasets combining spectral and weather information consistently outperform other data combinations. Validation results showed that the strongest numerical accuracy was observed for machine learning algorithms, e.g., random forest, with an *R^2^* of 0.9949, but it lacks spatial realism. On the other hand, DL models had comparable numerical accuracy and also produced smoother and more realistic spatial transitions but exhibited spatially structured residuals. To mitigate these spatial biases, residual kriging was applied to DL outputs, resulting in *RMSE* reduction of 35–45% and generating smoother pixel-level maps that preserved fine-scale heterogeneity and aligned with reported village yields. Moran’s I analysis confirmed significant residual spatial autocorrelation for DL models, justifying the use of geostatistical correction. Thus, the proposed hybridized framework emerged as best for balancing statistical accuracy with spatially realistic yield disaggregation. This study provides one of the first empirical demonstrations of village-to-pixel yield disaggregation using the identified weather and satellite band data combination.

## Introduction

The need for crop yield disaggregation stems from the limitations of aggregated yield statistics, which mask significant local variations in agricultural productivity, risk, and management practices. In many countries, including India, official crop yield estimates are typically reported at coarse administrative levels such as districts or states [1]. Although such aggregated statistics are valuable for regional production monitoring, they are inadequate for applications including precision agriculture, farm-level advisory services, and crop insurance schemes that require spatially explicit localized insights [2–5]. When yield data are aggregated, localized yield losses or gains cannot be accurately captured, leading to inefficiencies in resource allocation and increased basis risk in insurance payouts. Policy schemes such as crop insurance, subsidies, and disaster compensation also require farm-level yield estimates to reduce basis risk and ensure timely and transparent payouts to farmers [5,6]. Precision agriculture depends on field-level information to optimize input use, improve resource efficiency, and enhance farm profitability [3].

Beyond farm management and policy making, finer-scale yield estimates are increasingly important for food security monitoring and climate adaptation studies [4,7]. Disaggregating yield data to smaller geographical units, such as villages or farmer’s field, provides a more granular and realistic understanding of agricultural performance. To address these diverse needs, many studies reported disaggregation methods that translate coarse level yield data (*e.g.*, district) into finer-scale (*e.g.,* field) estimates [8], often referred to as a downscaling approach in some studies [9]. These methods are conceptually linked to spatial interpolation techniques [10].

**Fig 1:**
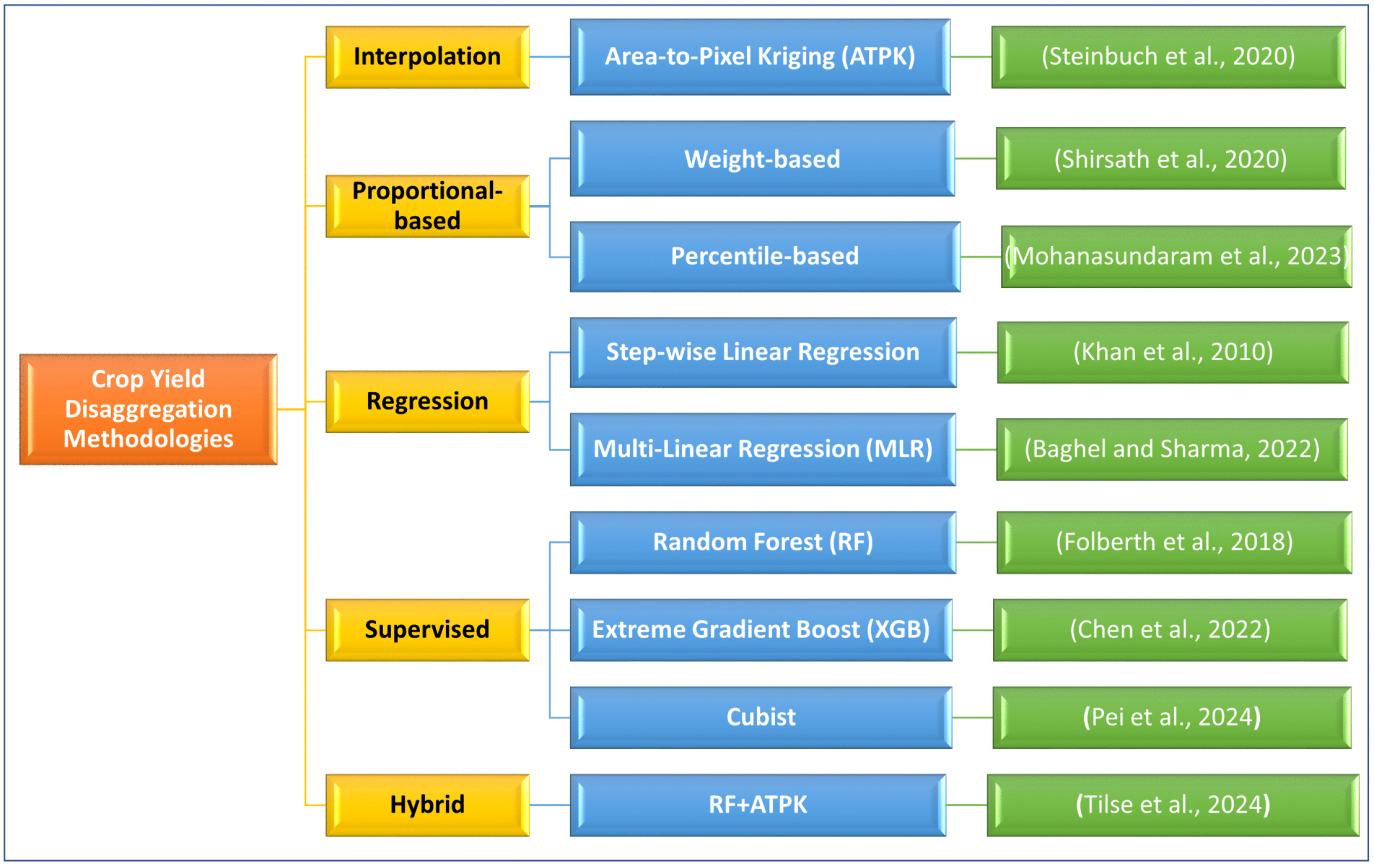
Representative crop yield disaggregation methodologies.

Existing yield disaggregation approaches can be broadly grouped into five categories (Fig 1). Geostatistical methods, particularly area-to-point kriging (ATPK), exploit spatial autocorrelation to interpolate fine-scale yield estimates from aggregated data [9,11]. However, ATPK cannot incorporate spectral or weather covariates, and the accuracy of kriging is highly sensitive to variogram specification [12] and sampling density [13,14]. Vegetation index (VI)-based allocation methods distribute aggregated yields across pixels with relative proportion [15]. While computationally simple and efficient, these methods assume linear proportionality between VIs and yield, often failing to capture temporal dynamics [16]. Regression-based frameworks relate yield variability to NDVI, climate, or biophysical covariates and have shown strong agreement with official statistics [17,18]. Nevertheless, regression approaches are typically linear, rarely account for nonlinear crop responses, and often ignore temporal variation within the growing season.

Recent advances in machine learning (ML) have enhanced the disaggregation of crop yield data by integrating multi-source environmental covariates and modeling complex nonlinear relationships. Ensemble ML models such as Random Forest (RF), Gradient Boosting (GB), and Extreme Gradient Boosting (XGB) have demonstrated strong performance in downscaling crop yields [19–21]. Although these models often achieve high numerical accuracy, they tend to produce spatial artifacts such as abrupt boundaries and block-like patterns due to the discrete nature of decision-tree structures, thereby limiting their spatial realism [22,23]. Here, spatial realism is defined as the model’s ability to capture and represent fine-scale heterogeneity and continuous spatial transitions in crop yield patterns.

As referred in previous paragraph, ML approaches significantly improve the accuracy of disaggregation, it is hypothesized that recently evolved Deep Learning (DL) approaches (*e.g.,* Long Short-Term Memory (LSTM), Gated Recurrent Units (GRU)), having strong capability in capturing nonlinear temporal dependencies, should further improve disaggregated yield values [24–27]. These studies have reported the use of the DL based models for improved yield prediction, but to the best of our knowledge, their application in yield disaggregation remains unexplored.

Parallel efforts in related domains reinforce the potential of multi-source downscaling. Early applications include remote sensing-based drought monitoring [28] and spatial interpolation for soil property mapping [13]. More recently, Mahmood et al. [29] demonstrated the value of ML for downscaling biophysical parameters, refining soil water indices from 1 km to 100 m resolution. These studies highlight the potential of disaggregation approaches but also reveal the scarcity of applications specifically targeting crop yield disaggregation.

Wheat and mustard are two of the most important crops in the rice-wheat cropping system of India, sustaining both household consumption and national food security [30]. Wheat occupies about 35.2 million hectares with a production of 128.5 million tons, while mustard covers approximately 9.9 million hectares with a production of 14.3 million tons [1]. Together, these crops contribute substantially to India’s agricultural economy and rural livelihoods [31]. However, their productivity is highly sensitive to weather variability and climate change. Even modest temperature increases have been shown to reduce wheat yields in India by 2–4% per degree Celsius rise, while heat stress and rainfall deficits further exacerbate risks during grain filling and maturity stages [32]. Mustard, similarly, is vulnerable to late-season heat stress and water scarcity, which can reduce oil content and yields [33].

With the above background, the present study proposes a hybrid framework that integrate DL algorithms and geostatistical methods to disaggregate village-level crop yields to pixel-level estimates. The study systematically evaluates traditional, ML, DL, and hybrid approaches for yield disaggregation; identifies an optimal minimum dataset combination comprising satellite spectral bands and weather variables for accurate yield disaggregation; and it provides one of the first empirical demonstrations of village-to-pixel yield disaggregation for wheat and mustard crops in semi-arid regions of India, balancing numerical accuracy with spatial realism.

### Study area

Haryana plays a central role in India’s food grain economy, ranking 2^nd^ nationally in mustard yield (after Gujarat) and 4^th^ in wheat production and area, while standing 3^rd^ in wheat yield (after Punjab and Chandigarh) [1]. The state is among the leading producers of wheat, rice, and mustard in India, with extensive irrigation coverage and relatively uniform management practices that reduce variability unrelated to biophysical conditions. Moreover, the availability of high-resolution remote sensing data such as Sentinel‑1 (Synthetic Aperture Radar) and Sentinel‑2 (multispectral optical imagery), obtained from the European Space Agency (ESA), with spatial resolutions of 10 m and 20 m depending on the bands [34]. Administrative boundaries [35], secondary yield data and ground truth data support from Krishi Vigyan Kendra, Adampur, Chaudhary Charan Singh Haryana Agricultural University, Hisar, and Department of Agriculture and Farmers Welfare Haryana, facilitated conduct of this study. Haryana’s robust data systems, coupled with its ongoing challenges related to water scarcity and crop diversification, make it an ideal site to develop and validate yield disaggregation models.

The study was conducted in Hisar and Bhiwani districts, located in the arid and semi-arid region of Haryana [36], which together provide a representative landscape of wheat and mustard cropping systems. Hisar is predominantly wheat-growing, with approximately 210 thousand hectares under wheat and 108 thousand hectares under mustard, whereas Bhiwani is mustard-dominated, with approximately 170 thousand hectares under mustard and 90 thousand hectares under wheat (Table 1). Five-year records (2018–2023) confirm the stability of crop dominance in the study area, with mustard consistently higher in Bhiwani and wheat dominating in Hisar (Table 1). The region with average annual rainfall of 350–500 mm, temperature ranges from 2–45°C [37], sandy loam to loam soils of medium fertility [38], and irrigation supplied mainly through tube wells and canals [39], all of which contribute to spatial variability in crop yields across village fields.

**Table 1:** Five-year records of wheat and mustard (2018-2023) from the Directorate of Economics and Statistics confirm this stability of crop dominance in the study area.

District	Year	Mustard Area (ha)	Yield (quintal/ha)	Wheat Area (ha)	Yield (quintal/ha)
Bhiwani	2018−19	129,900	18.2	113,000	44.2
	2019−20	142,300	15.1	107,400	41.9
	2020−21	169,600	17.4	116,330	42.4
	2021−22	169,596	17.6	86,914	42.7
	2022−23	171,430	13.4	90,640	41.0
Hisar	2018−19	76,000	21.4	221,100	49.6
	2019−20	74,400	18.2	224,300	45.6
	2020−21	73,500	19.0	231,250	49.7
	2021−22	107,032	19.2	206,860	46.4
	2022−23	108,120	19.3	208,130	49.9

**Fig 2:**
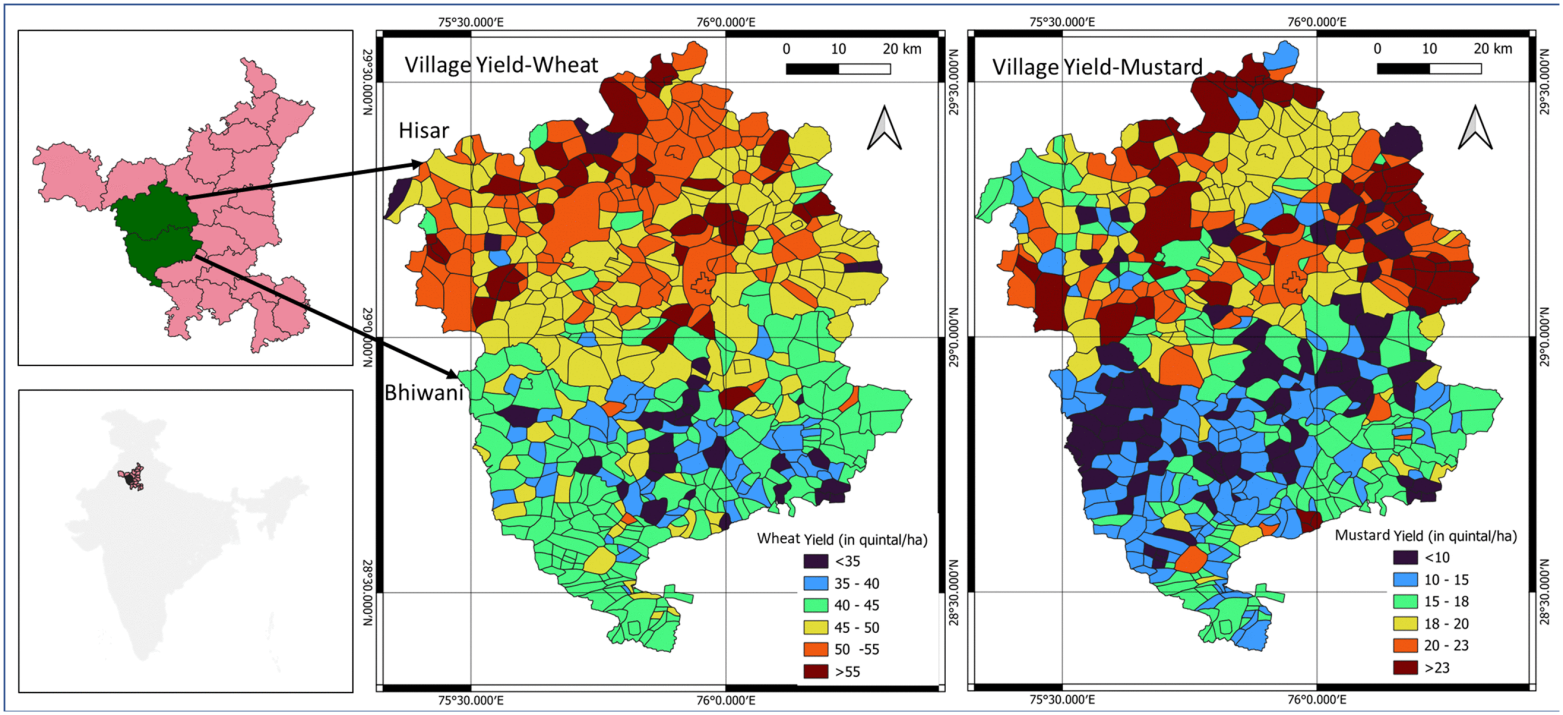
Study area and spatial distribution of village level wheat and mustard yields in Hisar and Bhiwani districts, Haryana, India, during the rabi season 2022−23. The spatial yield maps are author-generated outputs using QGIS. Village boundaries were obtained from Survey of India (SoI) village digital boundary shape files (Product Code: OVSF/-/10; https://onlinemaps.surveyofindia.gov.in/Digital_Product_Show.aspx; accessed Jan 2024).

Fig 2 illustrates the spatial distribution of wheat and mustard yields during the rabi season, classified into six categories for wheat (<35, 35–40, 40–45, 45–50, 50–55, > 55 quintals/ha) and mustard (<10, 10–15, 15–18, 18–20, 20–23, > 23 quintals/ha), highlighting the heterogeneity of yields across the landscape. This combination of temporal stability and spatial variability makes Hisar and Bhiwani ideal for evaluating village-to-pixel crop yield disaggregation approaches.

## Materials and methodology

This study developed a DL framework to disaggregate village-level crop yields into pixel-level estimates. The workflow comprised four sequential steps: (i) Dataset preparation, (ii) Regression and ML modelling for best inputs selection, (iii) DL modeling, and (iv) Geostatistical correction and validation. We used traditional disaggregation methods as benchmarking methods.

### Dataset preparation

This study integrated multi-source datasets (Table 2) covering crop yields, crop mask, weather, soil, and satellite observations to enable village-to-pixel disaggregation. Village-level yield statistics for wheat and mustard were obtained from the Department of Agriculture and Farmers Welfare, Haryana, and manually harmonized with administrative boundaries through village ID, block ID mapping, and cleaning were done in QGIS. Missing or inconsistent entries were corrected by imputing block-level averages, while villages absent from the shape file were excluded from analysis. We generated a crop mask using Sentinel-1 and Sentinel-2 data and validated it with crop ground truth (GT) collected from Hisar and Bhiwani districts. The crop mask was used to isolate cultivated pixels and ensure that spectral, radar, and weather information was extracted only from relevant crop areas.

**Table 2:** Overview of datasets used in the study.

Dataset	Source	Time * Period	Spatial Resolution	Description
**Crop Yield**	Department of Agriculture and Farmers Welfare, Haryana	Rabi	Village, block**	Wheat and mustard yields;
**Optical**	Sentinel-2 (ESA)	Nov, Dec, Feb	10-20 m	Bands- B2: Blue, B3: Green, B4: Red, B8: Near Infrared, B11: Shortwave infrared
**Radar**	Sentinel-1 (ESA)	Nov, Dec, Feb	10 m	Bands-VV, VH
**Weather**	ERA5-Land, ECMWF [40]	Nov-Mar	~27 km × 27 km	Air temperature (2 m), soil temperature (level 1), surface net solar radiation, total evaporation, total precipitation, leaf area index
**Soil**	SoilGrids250m [41]	Static	250 m	Bulk density, cation exchange capacity, coarse fragments, clay, sand, silt, nitrogen, organic carbon, pH (H₂O), soil organic carbon density
**Crop Mask**	Derived from Sentinel-2 + Sentinel-1 + ground truth	*	10 m	Wheat and mustard crop mask generated by the authors
**Shape file**	Survey of India [35]	Static	–	Spatial aggregation at village and block level

Note: *All data pertains to year 2022−23; ** village yield is used for disaggregation and both village and block for validation

Optical, radar, weather, and soil were accessed and downloaded via the Google Earth Engine (GEE) platform [42]. GEE provides cloud-based access to curated satellite archives and supports scalable geospatial processing, making it suitable for handling temporal and multi-sensor datasets required for this study [43]. All outputs were exported in GeoTIFF format for local processing and analysis.

Sentinel-2 optical bands (B2-Blue, B3-Green, B4-Red, B8-Near Infrared, and B11-Shortwave Infrared) and Sentinel-1 radar bands (VV, VH) were utilized to capture crop growth dynamics. Data were acquired for November, December, and February, corresponding to key growth stages of wheat and mustard during the rabi season. January imagery was excluded from the analysis due to persistently high cloud cover during this period, which limited the availability of cloud-free optical observations. March was excluded because most mustard harvesting occurs by late February or early March, reducing the relevance of spectral signals for yield estimation. All scenes were clipped to the study area boundary, mosaicked for multiple acquisition dates within each month, and averaged to generate a monthly composite for subsequent analysis.

Six weather features were extracted from the ERA5-Land Hourly dataset [40], produced by the European Centre for Medium-Range Weather Forecasts (ECMWF). Hourly records were aggregated into monthly means for the rabi season (November to March). These values were matched to village boundaries. Given the coarse spatial resolution (~27 km), multiple villages shared identical weather values, limiting village-scale differentiation, but they remained valuable when combined with higher-resolution satellite features. Soil properties were obtained from SoilGrids250m [41], based on over 230,000 soil profiles and ML predictions. Ten properties were aggregated to the village level and treated as static predictors.

**Fig 3:**
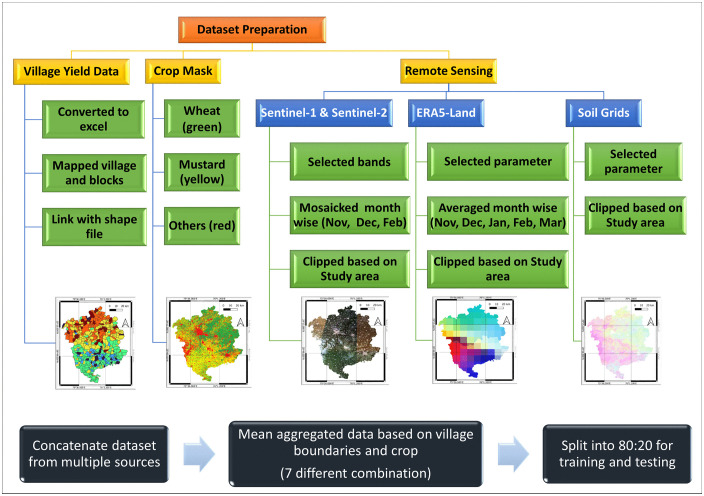
Dataset preparation and integration workflow for crop yield disaggregation, illustrating representative spatial layers for the Hisar and Bhiwani districts used in the study. The figure shows (from left to right) village administrative boundaries (Survey of India, Product Code: OVSF/-/10; https://onlinemaps.surveyofindia.gov.in/Digital_Product_Show.aspx; accessed Jan 2024) with yield, crop mask (wheat and mustard), Sentinel-2 RGB imagery, representative soil property raster’s (sand, silt, and clay), and selected weather raster’s (air temperature at 2 m, soil temperature level 1, and surface net solar radiation). The workflow diagram layout was created by the authors, and map visualizations were prepared in QGIS.

Village-level averages of weather, soil, and band information from GeoTIFF were extracted using a crop mask to isolate cultivated pixels and computed zonal statistics by using village boundaries. These feature extraction and aggregation were performed using Python-based geospatial libraries. The final analytical dataset consisted of 1,116 village-level samples (Fig 3). The dataset was randomly partitioned into training and validation subsets using an 80:20 split, resulting in 892 samples for model training and 224 samples for validation. The same data partitioning strategy was consistently applied across regression, ML, and DL models to ensure comparability of results. A schematic overview of the data preparation, feature extraction, and data partitioning workflow is presented in Fig 3, which also illustrates representative spatial layers including village boundaries, crop masks, satellite imagery, soil variables, and weather data.

Various datasets have been used in previous studies for crop yield prediction and disaggregation, including climate, satellite-derived VIs, and soil properties [21,24]. Different combinations used in different studies: NDVI combined with climatic data [18]; climate with leaf area index (LAI) [26]; climate and soil [19]; temporal LAI sequences [8,25]; VIs alone [9,15,16]; and climate-only datasets [2]. To assess the significance of one or multiple types of inputs, systematically combined to form seven combinations are summarized in Table 3.

**Table 3:** List of dataset combination and reference combination ID used in the study.

Dataset ID	Optical bands	Radar bands	Weather variables	Soil variables	Description
all-features	✓	✓	✓	✓	Full feature set
band-weather	✓	✓	✓	✗	Remote sensing + climate
band-soil	✓	✓	✗	✓	Remote sensing + soil
soil-weather	✗	✗	✓	✓	Environmental variables only
band-only	✓	✓	✗	✗	Remote sensing only
weather-only	✗	✗	✓	✗	Climate only
soil-only	✗	✗	✗	✓	Soil only

Note: Refer to Table 2 for a detailed description of optical, radar, weather, and soil variable.

### Algorithms

This study uses four categories of algorithms, namely traditional, regression, ML, DL, and hybrid, to assess the merits and demerits of each one (Table 4). Within each category, multiple algorithms have been used in this study. The algorithms have been used for various purposes like best dataset selection, model selection and visualization of the disaggregated yield values.

**Table 4:** List of algorithms used and reference Algo. ID used in the study.

Category	AlgoID	Algorithms	Purpose in this study
Traditional	Weight-based	Weight-based disaggregation [15]	Comparative analysis (model evaluation, metrics comparison, and visualization of pixel-level yield)
	Percentile-based	Percentile-based disaggregation [16]	
	ATPK	Area-to-point kriging [11]	Visualization of pixel-level yield
Regression	Linear	Multi Linear regression [18]	Dataset selection, algorithm selection, and comparative analysis
	Ridge	Ridge regression [44]	
	Lasso	Lasso regression [45]	
ML	RF	Random Forest [19]	
ML	GB	Gradient Boosting [46]	
ML	XGB	Extreme Gradient Boosting [20]	
DL	LSTM	Long Short-Term Memory [25]	Model building, Geostatistical correction, and comparative analysis
DL	GRU	Gated Recurrent Unit [27]	
Hybrid	LSTM-kriging	LSTM and ATPK Residual correction (proposed in this study)	
Hybrid	GRU-kriging	GRU and ATPK Residual correction (proposed in this study)	

Note: ML- Machine Learning; DL-Deep Learning

**Fig 4:**
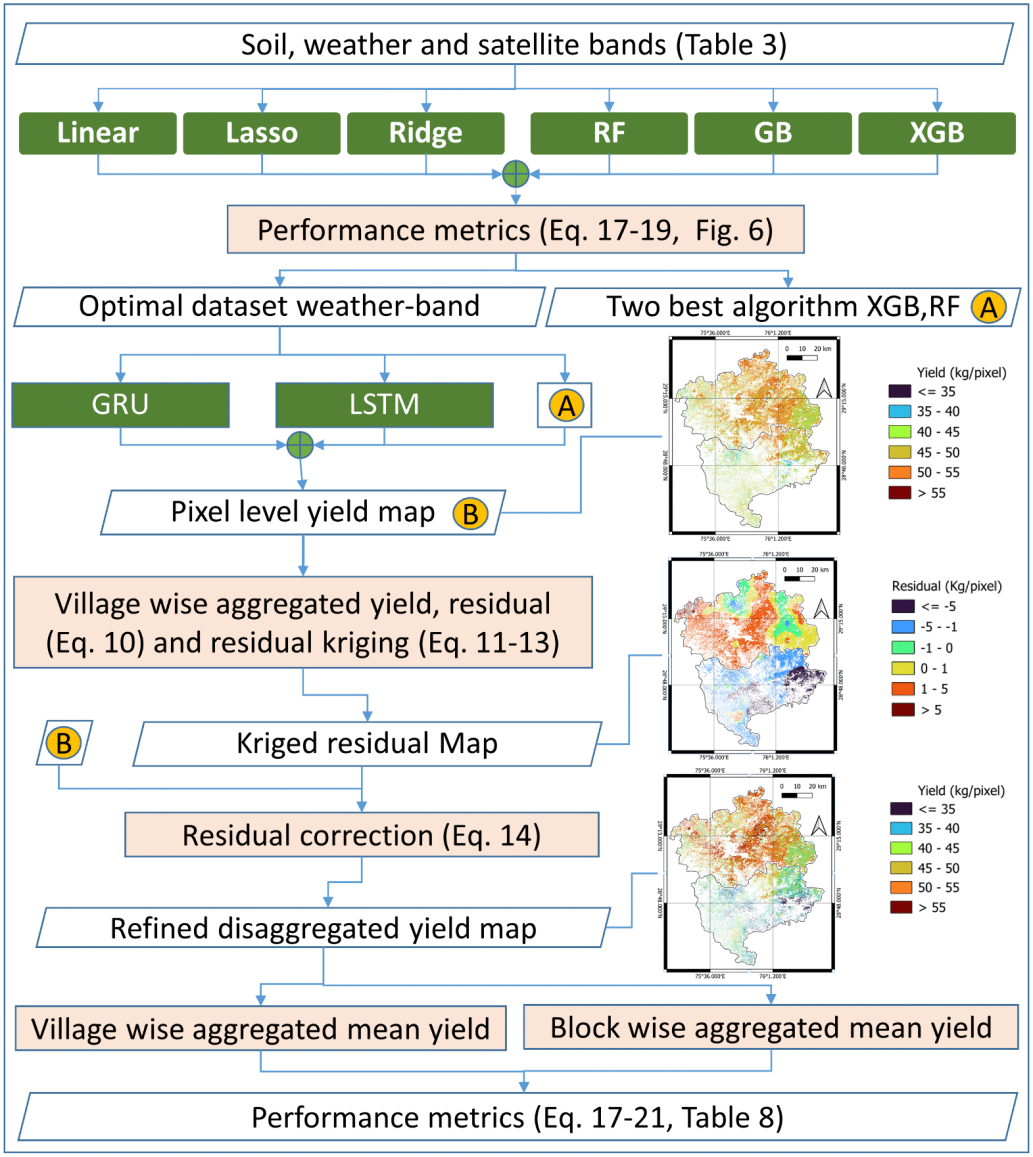
Flow diagram for village-to-pixel crop yield disaggregation. The yield prediction, residual, and corrected yield maps of Hisar and Bhiwani shown in the figure are author-generated (Python) and visualized using QGIS are included to illustrate the methodological process.

Fig 4, illustrates the hierarchical workflow of the proposed yield disaggregation framework. The first level corresponds to dataset and algorithm selection, where multiple feature combinations and predictive models are evaluated using quantitative performance metrics. The second level shows pixel-level yield prediction using selected ML and DL models. The third level depicts residual kriging, where spatial autocorrelation in model residuals is modeled using variogram analysis and in forth level applied to correct systematic spatial biases, resulting in the final disaggregated yield maps. In the final stage, corrected yields are aggregated to administrative units and model performance is evaluated.

### Dataset selection using regression and ML models

The first stage of analysis employed regression and ML to identify the best dataset combination among all seven datasets (refer to Table 3, Fig 4). This stage was designed to screen input feature combinations and select a consistently performing dataset prior to DL based pixel-level yield modeling. Regression and ML models are well suited for capturing nonlinear interactions among spectral, weather, and soil variables at the village level while maintaining computational efficiency.

Linear regression (Linear) assumes linear dependence between predictors and yield [47]:


$$y=\beta_{\text{0}}+\sum_{i=\text{1}}^{n}\beta_{\text{i}}x_{\text{i}}+\varepsilon$$ (1)

where 𝑦 is the predicted yield, 𝑥_𝑖_ are the features, 𝛽_0_ is the intercept, 𝛽_𝑖_ are the coefficients, and 𝜖 is the error term.

Ridge regression (Ridge) adds L2 regularization to handle multicollinearity [48]:


$$min\;\Sigma {\left(y_{\text{i}}-{\hat{y}}_{\text{i}}\right)}^{\text{2}}+\lambda \;\Sigma {\beta_{\text{j}}}^{\text{2}}$$ (2)

where *𝜆* is the regularization parameter controlling the penalty term. Ridge Regression shrinks the coefficients but does not set them exactly to zero.

Lasso regression (Lasso) extends linear regression with L1 regularization, which can shrink some coefficients to zero, thereby performing variable selection [49]:


$${\text{min}\Sigma \left(y_{\text{i}}-{\hat{y}}_{\text{i}}\right)}^{\text{2}}+\lambda \;\Sigma \left|\beta_{\text{j}}\right|$$ (3)

Random Forest (RF) is an ensemble learning algorithm that builds multiple decision trees using bootstrap samples and averages their predictions to reduce variance [50].


$${\hat{y}}_{\text{i}}=\frac{\text{1}}{T}\sum_{t=\text{1}}^{T}f_{t}(x)$$ (4)

where, *f*_*t*_ *(x)* = prediction from the *t*^*th*^ decision tree, *T* = total number of trees. Each tree *f*_*t*_ is trained on a bootstrap sample of the data.

Gradient Boosting (GB) constructs an additive model by sequentially fitting decision trees to the residual errors of previous trees, minimizing a differentiable loss function via gradient descent [51].


$${\hat{y}}_{\text{i}}=\sum_{t=\text{1}}^{T}{\eta f}_{t}(x_{i})$$ (5)

where, *T* = total number of trees (boosting iterations), *η* = learning rate

Extreme Gradient Boosting (XGB) improves upon traditional gradient boosting by incorporating second-order derivatives in the optimization process and adding regularization terms to control model complexity [52].


$${\hat{y}}_{\text{i}}=\sum_{t=\text{1}}^{T}f_{t}(x_{i}),f_{t}\in F$$ (6)

Where *F* = space of all possible trees

Linear, Ridge, Lasso, RF, GB, and XGB models were trained using village-level yield observations. Model performance was evaluated using prediction error metrics, and the dataset-model combination yielding the lowest error was selected as the optimal input configuration for subsequent DL modeling. The best-performing regression and ML models were also retained for comparison with DL and hybrid DL-kriging approaches.

## DL model

**Fig 5:**
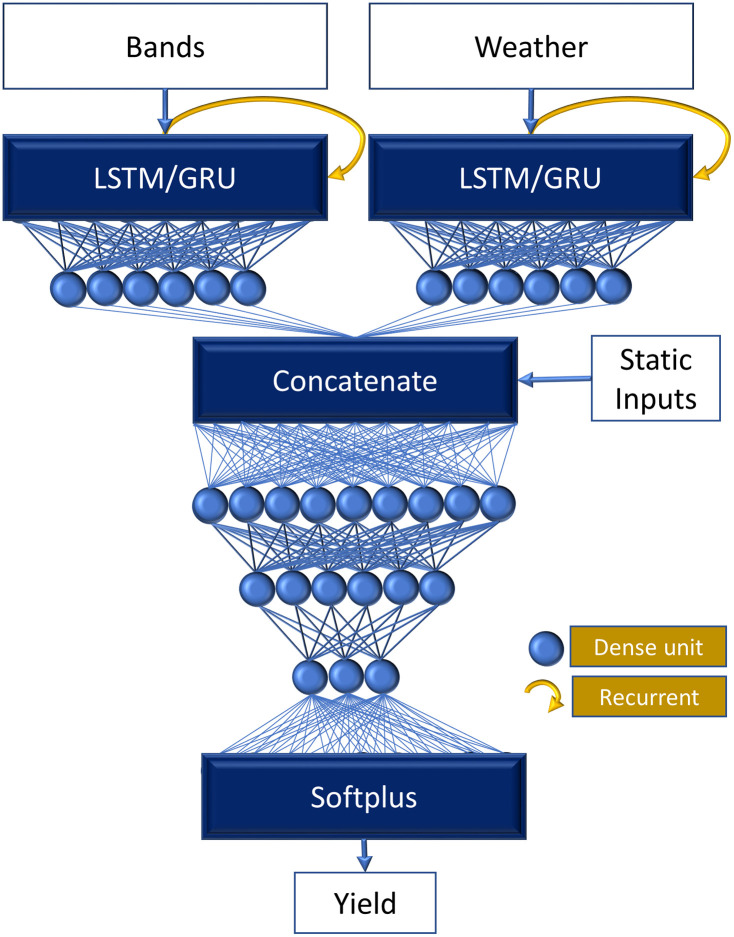
DL based yield disaggregation model for temporal and static inputs. GRU and LSTM models share identical architectures, differing only in the recurrent cell type.

Using the optimal dataset combination identified in the previous stage, Recurrent Neural Networks (RNN) algorithm were implemented which capture temporal dependencies and generate pixel-level yield predictions. Two RNN architectures were implemented: Long Short-Term Memory (LSTM) [53] and Gated Recurrent Unit (GRU) [54]. Both models share the same architecture, differing only in the internal recurrent structure. The LSTM model comprised 25,889 trainable parameters, while the GRU model comprised 23,553 trainable parameters. This DL model integrates three types of inputs (Fig 5):

Bands inputs 𝑋_bands_ ∈ 𝑅 ^𝑅1× 𝐹1^: time-series (T1=3) of backscatter and spectral bands (F1=7).

Weather inputs 𝑋_weather_ ∈ 𝑅^𝑅2×𝐹2^: time-series(T2=5) of weather variables (F2=6).

Static inputs 𝑋_static_ ∈ 𝑅^𝐹3^: features such as soil or crop type (F3=1).

Each sequential input is processed through its own recurrent module (either GRU or LSTM) with 32 hidden units, followed by a Dense layer of 16 neurons activated by ReLU:


$$h_{t}=\left\{ \begin{array}{c} GRU\left(x_{t},h_{t-\text{1}}\right)\;for\;GRU\;Model\\ LSTM(xt,ht-\text{1},ct-\text{1})\;for\;LSTM\;model \end{array}\right.\;$$ (7)

LSTM maintains long-term cell state 𝐶_𝑡_ and hidden state ℎ_𝑡_ via input, forget, and output gates. GRU architectures simplify recurrence with update and reset gates directly controlling hidden state transitions.

The static input branch bypasses temporal processing, preserving its spatial and categorical characteristics. Outputs from the bands, weather, and static branches are concatenated to form a unified feature vector $X_{merge}=[h_{bands},h_{weather},X_{static}]$. This merged representation is passed through three fully connected layers (128, 64, and 32 neurons) with ReLU activations to learn complex nonlinear relationships:


$$X_{d\text{3}}=\text{ReLU}(W_{\text{3}}\times \text{ReLU}(W_{\text{2}}\times \text{ReLU}(W_{\text{1}}\times X_{merge}+\text{b1})+\text{b2})+\text{b3})$$ (8)

Where, *W*_*1*_, *W*_*2*_, and *W*_*3*_ denote the learnable weight matrices and *b*_*1*_, *b*_*2*_ and *b*_*3*_ represent the corresponding bias vectors of the successive fully connected layers, while ReLU is the nonlinear activation function applied at each layer.

Finally, a Softplus activation function is applied at the output layer to ensure non-negative yield predictions suitable for regression tasks [55].


$$\hat{y}=\text{log}(\text{1}+e^{W_{\text{0}}X_{d\text{3}}+b_{\text{0}}})$$ (9)

Mean squared error was used as the optimization loss, while mean absolute error was monitored during training to assess convergence and model stability [56,57]. Regularization was achieved implicitly through limited network depth, early stopping, and an 80:20 training-validation split. Training was conducted for a maximum of 500 epochs with early stopping patience set to 25 and a batch size of 32.

Table 5 shows the hyper parameter setting for different ML and DL methods. All models were trained on village-level data, applied to pixel-level features for yield prediction, and aggregated back to village-level yields to estimate the residual for further geostatistical correction.

**Table 5:** Hyper parameter settings used for model implementation.

Model group	Hyper parameters	Values
Tree-based models (RF, GB, XGB)	Number of trees (n_estimators)	100
	Learning rate	0.1 (GB, XGB only)
	Bootstrap sampling	Enabled (RF only)
	Random seed	42
Recurrent DL models (GRU, LSTM)	Recurrent units (band branches)	32
	Recurrent units (weather branches)	32
	Dense units after recurrent layers	16
	Fully connected layers	128 − 64 − 32
	Dropout rate	0.1
	Activation functions	ReLU (hidden), Softplus (output)
	Optimizer/ Loss	Adam/ MSE
	Monitoring metric	Mean absolute error

### Geostatistical correction

Although regression, ML, and DL models captured feature-yield relationships, their residuals exhibited spatial autocorrelation. To address this, a robust regression-kriging framework [58] was applied to refine the results. Residuals for each village 𝑖 were computed as:


$$e_{i}=y_{\text{i}}-{\hat{y}}_{\text{i}}$$ (10)

where 𝑦_𝑖_ is the observed village yield and ${\hat{y}}_{\text{i}}$ is the predicted mean yield aggregated from ML/DL maps. Village polygons were converted to centroids, and semi-variograms [12] were fitted to model spatial dependence:


$$\gamma (\text{h})=\frac{\text{1}}{\text{2}N(h)}\sum_{i=\text{1}}^{N(h)}{\left[e\left(s_{i}\right)-e(s_{i}+h)\right]}^{\text{2}}$$ (11)

where ℎ is the separation distance, 𝑁(ℎ) is the number of point pairs at distance ℎ, and 𝑒(𝑠_𝑖_) is the residual at location 𝑠_𝑖_. Residuals were interpolated using kriging with the best-fitting variogram model (‘linear,’ ‘spherical,’ ‘Gaussian,’ or ‘exponential’) selected through cross-validation.

In kriging, residuals were modeled as a weighted sum of nearby observations [59]:


$$e_{krig}(s)=\sum \lambda_{i}e\left(s_{i}\right)$$ (12)

where 𝜆_𝑖_ are kriging weights assigned to each residual 𝑒(𝑠_𝑖_).

The weights depend on the variogram model: closer points (small 𝛾(ℎ)) get higher weights, while distant or weakly correlated points get smaller weights. 𝜆_𝑖_ are derived from solving this kriging matrix equation using *γ(h)*.


$$\sum_{j=\text{1}}^{n}\lambda_{j}\gamma ({\text{s}}_{i}-{\text{s}}_{j})+\mu =\gamma ({\text{s}}_{i}-{\text{s}}_{\text{0}}),\text{i}=\text{1},...,\text{n}$$ (13)

*γ(s*_*i*_ *– s*_*j*_*)*: semi variance between observed locations *s*_*i*_ and *s*_*j*_*, γ(s*_*i*_ *– s*_*0*_*)*: semi variance between observed location *s*_*i*_ and the prediction location *s*_*0*_, μ: Lagrange multiplier enforcing unbiasedness.

The resulting kriging residual surface was then added back to the predicted raster. [58]:


$$y^{*}(s)=\hat{y}(s)+e_{krig}(s)$$ (14)

where $y^{*}(s)$ is the corrected yield at location 𝑠. This refinement improved fine-scale accuracy by combining feature-driven predictions with spatially dependent corrections. Block-level aggregations of corrected predictions were validated against reported statistics. Performance of DL- kriging was benchmarked against the best standalone DL and ML model under identical dataset combinations.

### Traditional methods

The three commonly used traditional disaggregation methods, namely weight-based, percentile-based, and area-to-point kriging (ATPK), were implemented as benchmarking methods.

Weight-based disaggregation [15]: Pixel-wise yield was calculated as:


$$Yield=\frac{{NDVI}_{P}}{\sum_{i=\text{1}}^{p}{NDVI}_{i}}X\;P$$ (15)

where *NDVIₚ* is the NDVI value for pixel *p*, *P* is the reported production for the administrative unit.

Percentile-based disaggregation [16]: Yield values were scaled as:


$$Yield=\frac{Maxyield-Minyield}{{MaxNDVI}_{\text{9}\text{5}}-{MinNDVI}_{\text{5}}}X\;{NDVI}_{p}$$ (16)

where *MaxNDVI₉₅* and *MinNDVI₅* are the 95^th^ and 5^th^ percentile NDVI values, respectively.

Area-to-Point Kriging: village-level yields were disaggregated to pixel scale without external covariates [11,59]. These methods were not part of the main workflow but served as external benchmarks against ML, DL, and residual kriging approaches.

### Model evaluation

Model performance was assessed using the coefficient of determination (*R*^2^), adjusted coefficient of determination (*adjusted R^2^*), Root Mean Squared Error (*RMSE*), Mean Absolute Error (*MAE*), and Mean Absolute Percentage Error (*MAPE*). Additionally, the statistical significance of differences among different dataset models was assessed using the Friedman chi-square test, followed by post-hoc analysis.

The coefficient of determination (*R*^2^) [60] was computed as:


$$R^{\text{2}}=\text{1}-\frac{\Sigma (y_{\text{i}}-{\hat{y}}_{\text{i}}{)}^{\text{2}}}{\Sigma (y_{\text{i}}-\bar{y}{)}^{\text{2}}}$$ (17)

where *yᵢ* represents observed yield, *ŷᵢ* is the predicted yield, and *ȳ* is the mean of observed yields.

The adjusted coefficient of determination (*adjusted R^2^*) accounts for the number of predictors relative to the number of observations and penalizes model complexity. It was computed as [61]:


$$\text{Adjusted }R^{\text{2}}=\text{1}-\left(\frac{n-\text{1}}{n-p-\text{1}}\right)\left(\text{1}-R^{\text{2}}\right)$$ (18)

where *n* is the number of observations and *p* is the number of predictors.

The Root Mean Squared Error (*RMSE*) [56] was computed as:


$$RMSE=\sqrt{\frac{\text{1}}{n}\times \Sigma (y_{\text{i}}-{\hat{y}}_{\text{i}}{)}^{\text{2}}}$$ (19)

where n is the number of observations. *RMSE* measures the average magnitude of prediction error, with lower values indicating better model performance.

The Mean Absolute Error (*MAE*) was computed as [56]:


$$MAE=\frac{\text{1}}{n}\times \Sigma \left|y_{\text{i}}-{\hat{y}}_{\text{i}}\right|$$ (20)

*MAE* represents the average absolute difference between observed and predicted values, providing a straightforward measure of prediction accuracy.

The Mean Absolute Percentage Error (*MAPE*) was computed as [57]:


$$MAPE=\frac{\text{1}\text{0}\text{0}}{n}\sum \left|\frac{y_{\text{i}}-{\hat{y}}_{\text{i}}}{y_{\text{i}}}\right|$$ (21)

*MAPE* expresses prediction accuracy as a percentage, allowing for easier interpretation of model error relative to the magnitude of the observed values.

The Friedman chi-square test [62] was applied to assess whether performance differences among models were statistically significant across datasets. The test statistic is given by:


$$\chi_{\text{F}}^{\text{2}}=\frac{\text{1}\text{2}N}{k(k+\text{1})}\sum R_{j}^{\text{2}}-\frac{k{\left(k+\text{1}\right)}^{\text{2}}}{\text{4}}$$ (22)

where 𝑁 is the number of dataset, 𝑘 is the number of model, and 𝑅_𝑗_ is the average rank of model 𝑗.

When significant differences were detected, pairwise comparisons were conducted using the Nemenyi post-hoc test [63]. The critical difference (*CD*) was computed as:


$$CD=q_{\alpha }\times \sqrt{\frac{k(k+\text{1})}{\text{6}N}}$$ (23)

where *q*_*α*_ is the critical value of the Studentized range statistic for significance level *α*, *k* is the number of models compared, and *N* is the number of datasets. If the average rank difference between two models exceeds *CD*, their performance difference is considered statistically significant.

Spatial autocorrelation in the predicted yield residuals was quantified using Moran’s I statistic [64]. Moran’s I was computed using a k-nearest neighbor spatial weights matrix (k = 8) constructed from pixel-level prediction locations in projected coordinate space. Positive Moran’s I values indicate spatial clustering of similar residuals, while values near zero indicate spatial randomness and negative values indicate spatial dispersion.

### Software and computational environment

All spatial and statistical analyses were conducted in Anaconda Jupyter Notebook using a Python 3.8.20 environment on a workstation equipped with an NVIDIA RTX A5000 GPU (24 GB memory). This setup provided sufficient computational capacity for DL model training, geostatistical processing, and large raster handling.

Geospatial data preprocessing was performed with geopandas, rasterio, rasterstats, and shapely for handling vector and raster layers, including resampling and zonal aggregation. Regression and ML models were implemented with scikit-learn and xgboost, while DL models were built with TensorFlow and Keras. For geostatistical corrections, pykrige was used for variogram fitting and kriging, with pyproj handling coordinate reference systems and scikit-learn providing K-fold cross-validation. Visualization and statistical analyses were supported by matplotlib, seaborn, and scipy. stats, while scikit-posthocs enabled post-hoc significance testing.

## Result and discussion

This section presents the outcome of the proposed hybrid village-to-pixel yield disaggregation methodology and its counterparts namely traditional, ML and DL. As a byproduct, the study also evaluates the use of various data sources combination and filters to the optimum dataset features for yield disaggregation. The first part present results of experiments related to the identification of the optimum dataset and algorithms for yield disaggregation. Then discusses disaggregation on a selected dataset across the algorithms and also presents the disaggregated results in the map.

### Dataset selection

**Fig 6:**
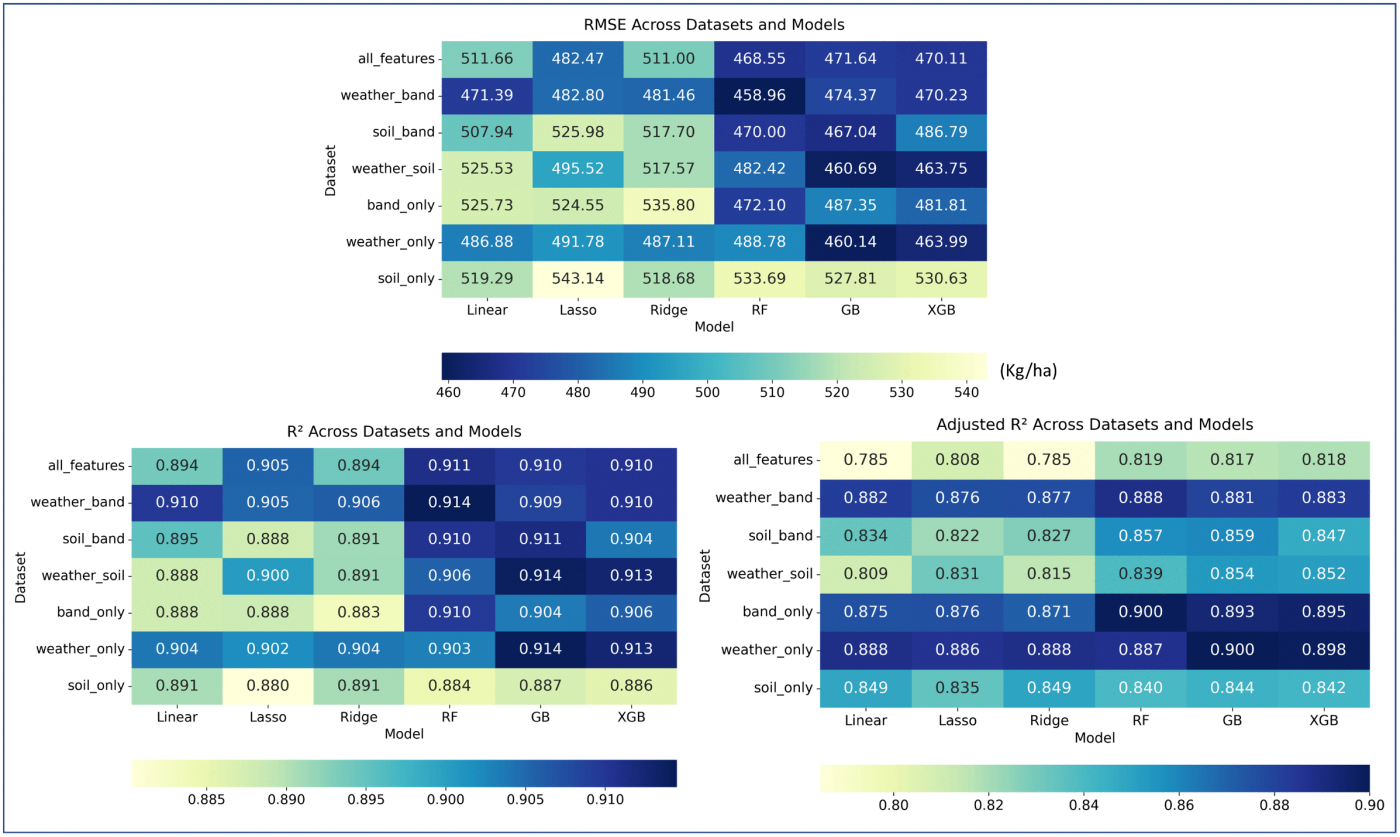
RMSE, R^2^ and adjusted R^2^ across datasets and models for test data.

Fig 6 presents the performance metrics, namely *R*^*2*^*, adjusted R*^*2*^ *and RMSE* and in a 7 x 6 grid (datasets x model) across datasets and model. A horizontal bar at bottom represents the color scale with corresponding values. It is observed that the datasets *band-weather*, *weather-only*, and *all-features* produced the higher *R*^*2*^ and lower *RMSE* values across most of the models. However, adjusted *R^2^* reveals a different pattern, it explicitly penalizes model complexity and redundant predictors. As a result, datasets with fewer but more informative variables are favored. In this context, the *adjusted R^2^* values indicate that weather-only, band-only, and band-weather outperform the all-features dataset, highlighting the limited marginal benefit of including additional predictors when they do not contribute proportionally to explanatory power.

**Fig 7:**
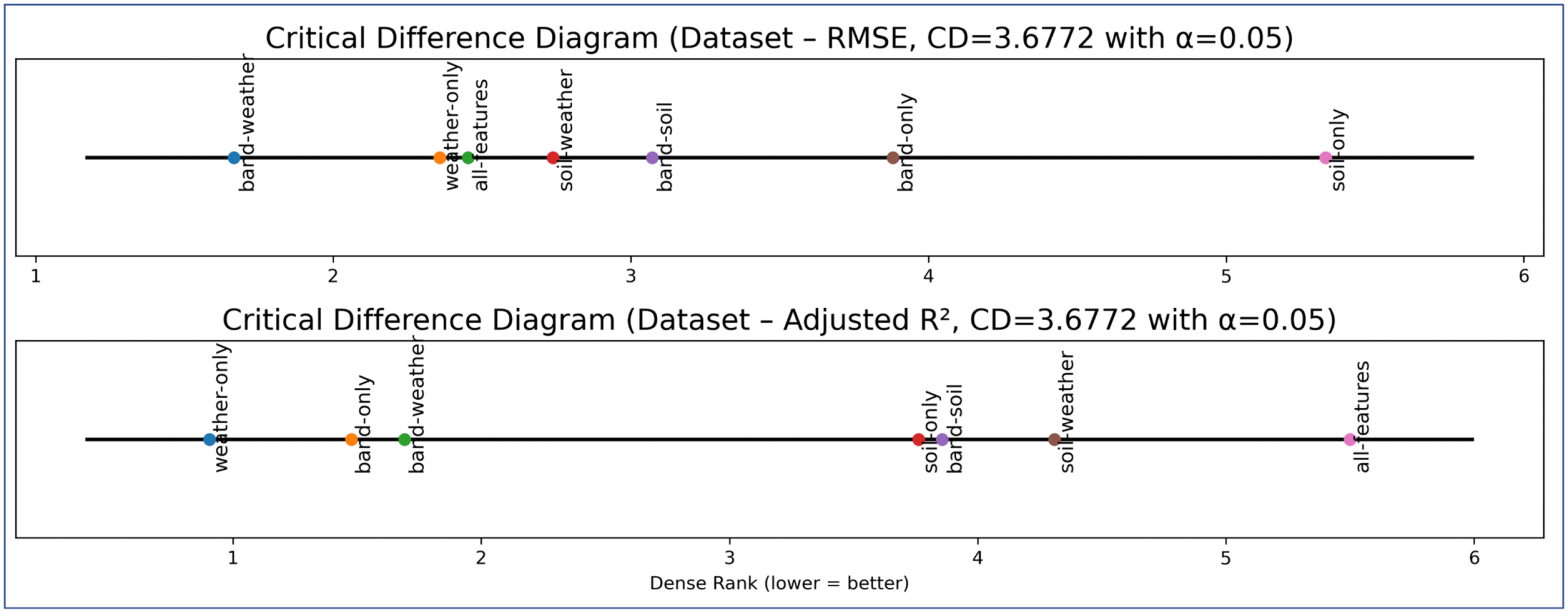
Comparison of the average rank of different datasets for testing the significance based on *RMSE* and *adjusted R^2^.*

To further test whether the dataset results are significantly different, Friedman tests were conducted on values of *RMSE* (*χ*^*2*^ = 19.50; *p* = 0.0034) and *R^2^* (*χ*^*2*^ = 19.68; *p* = 0.00315), confirming significant variation among datasets. Further Post-hoc analysis further identified that the soil-only dataset performed significantly worse (average rank difference < *CD* = 3.6772) than both *band-weather* and *weather-only* datasets. No other pairwise differences reached significance. These results are summarized in Table 6 and visually illustrated in Fig 7.

**Table 6:** Comparison of average rank difference among different datasets.

Dataset	*band-weather*	*weather-only*	*all-features*	*soil-weather*	*band-soil*	*band-only*
*band-weather*	0					
*weather-only*	0.34	0				
*all-features*	0.5	0.16	0			
*soil-weather*	1.34	1	0.84	0		
*band-soil*	2	1.66	1.5	0.66	0	
*band-only*	3.34	3	2.84	2	1.34	0
*soil-only*	4.17	3.83	3.67	2.83	2.17	0.83

**Note**: critical difference = 3.6772 for significance level α = 0.05

As shown in Fig 7a-b, the *weather-only* dataset yielded deceptively low *RMSE* and strong *adjusted R^2^* values due to the coarse resolution of ERA5-Land weather data used in this study, which assigns identical weather values to many villages and fails to capture fine-scale heterogeneity. This artificially reduces prediction variance and inflates performance, and thus these results should not be interpreted as evidence that weather (*weather-only*) alone is sufficient. The *soil-only* dataset performed poorly (*RMSE* > 5.20 q/ha, *R*^*2*^ < 0.89 across models), as confirmed by post-hoc tests (Table 6), reflecting the limited explanatory power of static soil variables for intra-season yield variation.

In all features, when soil information was added to other predictors (weather and band), performance did not improve; in fact, accuracy slightly decreased compared with band-weather. A paired t-test between band-weather and all-features also confirmed no significant difference (*p* = 0.145). Integrating insights from adjusted *R^2^*, results indicate that while all-features achieves strong raw performance, it does so at the cost of increased complexity without commensurate gains in explanatory power. Although band-only achieved high adjusted *R*^*2*^, it lacks explicit meteorological information and did not perform *RMSE* or *R^2^* well as compared to band-weather. Therefore, band-weather was selected as the final dataset combination for DL modeling and comparison.

### Algorithm selection

The set of algorithms evaluated in this study was selected to represent a broad spectrum of modeling paradigms commonly used in yield prediction and spatial modeling studies. Linear regression, Ridge, and Lasso were included as baseline parametric models to assess the limits of linear relationships between predictors and yield. Tree-based ensemble methods, namely RF, GB, and XGB, were chosen due to their proven ability to model nonlinear interactions and handle multi-source remote sensing and environmental data. This diverse selection allows a systematic comparison of simple, regularized, and nonlinear ensemble models before advancing to DL and hybrid geostatistical approaches.

As per Fig 6, among the algorithms, RF performed particularly well, achieving *RMSE* below 4.69 q/ha while maintaining high *R*^*2*^ values (>0.91). XGB and GB also performed competitively, though RF remained more stable across datasets. In contrast, linear models (Linear, Ridge, Lasso) generally yielded higher *RMSE* values.

**Fig 8:**
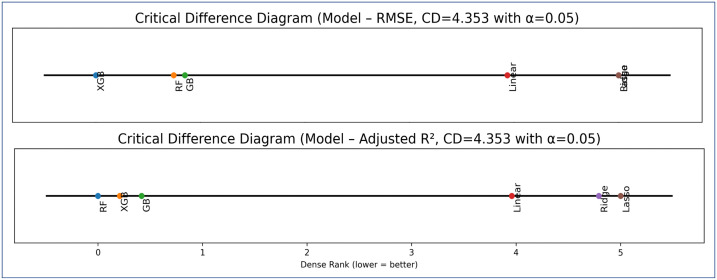
Comparison of the average rank of different models for testing the significance based on RMSE.

Friedman test *RMSE* across all six models (*χ*^*2*^ = 7.95; *p* = 0.159) revealed no statistically significant differences. Nevertheless, average ranks analysis (Fig 8) indicated that tree-based ensemble models XGB, RF, and GB outperformed the regression models. Considering both performance stability across dataset and relative ranking based on *RMSE* and *adjusted R^2^*, XGB and RF were selected for subsequent analysis. These models were retained as strong and widely used ML baselines for comparison with DL and kriging-based hybrid approaches.

### DL model performance

Using the band-weather dataset, DL models were trained to capture yield dependencies on temporal values of weather and bands.

**Fig 9:**
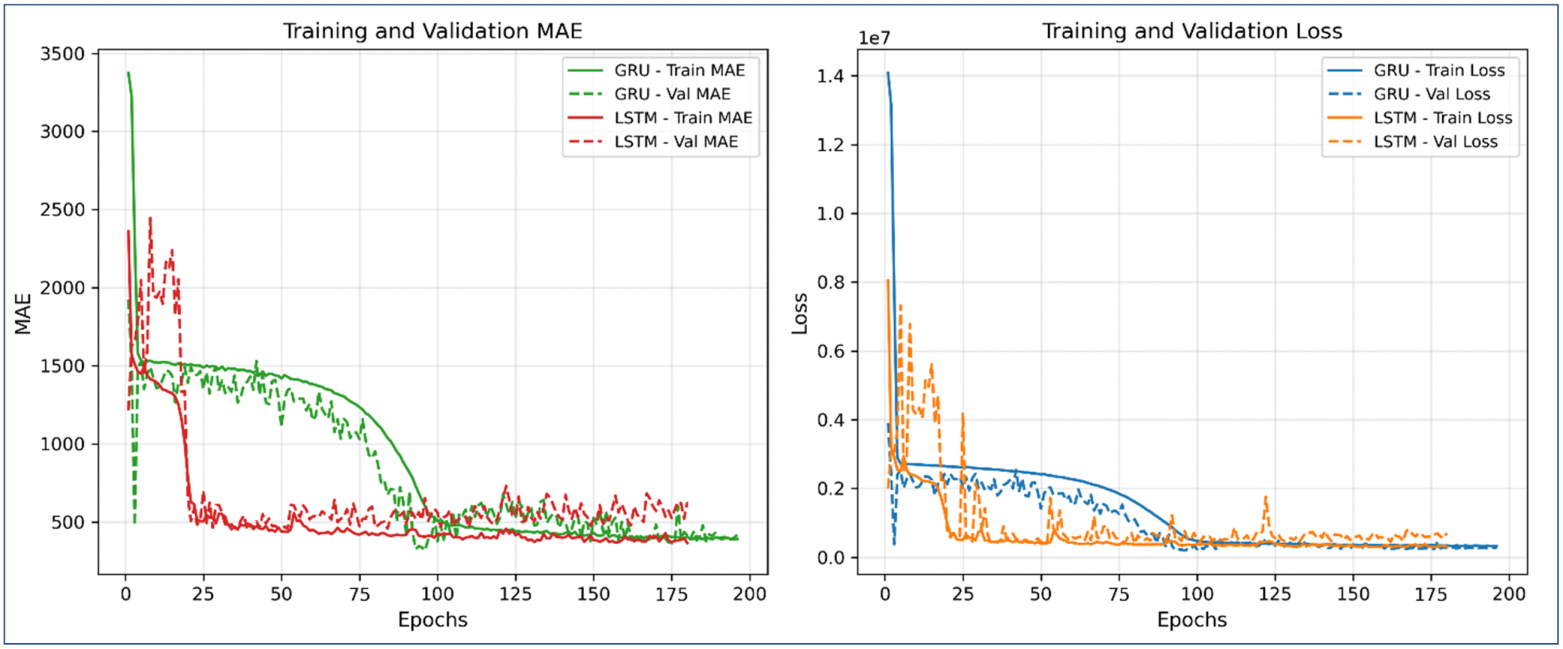
Comparison training and validation *MAE* and loss for LSTM and GRU models.

Fig 9 illustrates the training and validation *MAE* (left) and loss (right) curves for both models. The LSTM exhibited faster and more stable convergence, with *MAE* and loss stabilizing within the first 30–40 epochs, reflecting efficient learning and reduced overfitting. In contrast, the GRU model showed a delayed convergence pattern, requiring more epochs to reach optimal performance, though it eventually achieved comparable validation loss. This difference can be attributed to GRU’s simpler gating mechanism, which can sometimes require longer adaptation when handling complex temporal dependencies.

The LSTM model achieved an *RMSE* of 4.89 q/ha and an *R*^*2*^ of 0.9013, whereas the GRU model achieved a slightly better *RMSE* of 4.75 q/ha and *R*^*2*^ of 0.9024, indicating that both performed comparably with ensemble ML models.

### Spatial visualization of disaggregated yield

This section presents a comparative visual assessment of the spatial patterns generated by different yield disaggregation approaches. Spatial visualization is used to evaluate the realism, continuity, and artifact behavior of the disaggregated yield maps beyond quantitative accuracy metrics. The analysis is organized to reflect the methodological workflow, progressing from pre-kriging results to residual kriging and post-kriging outcomes.

### Traditional disaggregation

**Fig 10:**
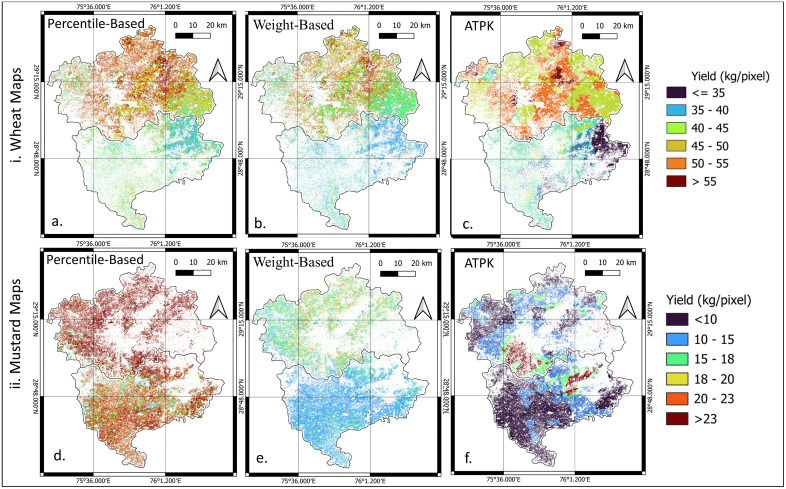
Comparison among traditional yield disaggregation methods for Hisar and Bhiwani districts: i. wheat map and ii. mustard map. These maps are analytical raster outputs (Python).

The traditional approaches were presented without any spatial correction to serve as baseline methods. The weight-based and percentile-based, showed limited ability to reproduce fine-scale spatial heterogeneity (Fig 10(a), 10(b), 10(d) and 10(e)). These methods rely on temporally aggregated average or a peak VI values for the season derived from different discrete acquisition periods. As a result, the generated yield maps exhibited spatially distinct yield zones driven by VI temporal grouping rather than true yield variability, thereby producing artificial partitions across the landscape [65]. Thus, we observed three sets of partitions in yield maps (left-light green, middle-orange, brown, and right-parrot green) corresponding to different VI acquisition periods. Area to Point Kriging of village level yields provided a smooth reference surface but lacked spatial realism, as it could not leverage spectral or weather covariates necessary to capture fine-scale yield variability (Fig 10(c) and 10(f)).

### ML and DL yield disaggregation

**Fig 11:**
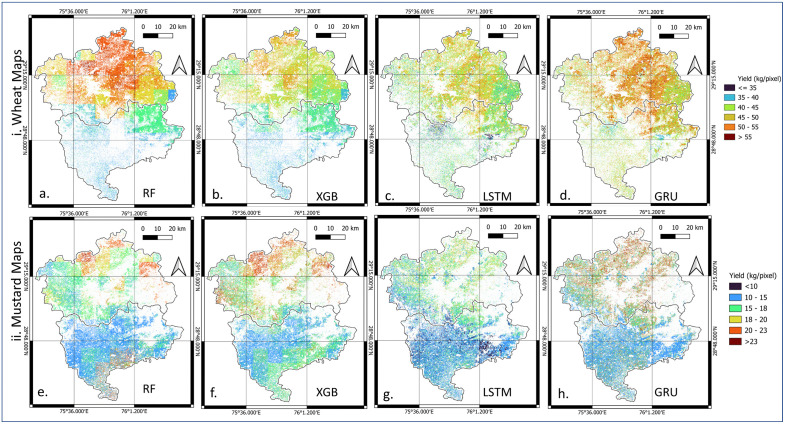
Comparison among different ML, and DL yield disaggregation methods for Hisar and Bhiwani districts: i. wheat map and ii. mustard map. These maps are analytical raster outputs (Python) produced in this study and visualized using QGIS.

Spatial visualization of ML- and DL-based disaggregated yield maps revealed clear differences in spatial structure and realism (Fig 11). ML-based maps (Fig 11(a-b) and 11(e-f)) showed sharp contrasts and exaggerated block boundaries, resulting in spatial discontinuities and linear artifacts due to decision tree splits [22,23]. In contrast, the DL models (LSTM, GRU) generated visually smoother and more coherent yield patterns, better representing gradual yield transitions across the landscape (Fig 11(c-d) and 11(g-h)). Between these two DL models, GRU maps displayed stronger structural coherence and preserved within-village variability without over-smoothing.

### Residual kriging and variogram model selection

To account for spatial dependence in model residuals, multiple variogram models (linear, spherical, Gaussian, and exponential) were evaluated using cross-validation. The cross-validation *RMSE* values and the corresponding selected variogram models are summarized in Table 7.

**Table 7:** Cross-validation *RMSE* (quintals ha^−1^) for candidate variogram models and selected variogram type for residual kriging across crops, districts, and predictive models.

Model	District	Crop	Linear	Spherical	Gaussian	Exponential	Selected
RF	Hisar	Wheat	4.808	4.820	4.835	4.836	Linear
RF	Hisar	Mustard	3.756	3.835	3.777	3.764	Linear
RF	Bhiwani	Wheat	5.672	5.880	5.899	5.845	Linear
RF	Bhiwani	Mustard	3.052	3.036	3.066	3.026	Exponential
XGB	Hisar	Wheat	4.826	4.847	4.847	4.882	Linear
XGB	Hisar	Mustard	3.906	3.937	3.898	3.900	Gaussian
XGB	Bhiwani	Wheat	5.657	5.825	5.842	5.773	Linear
XGB	Bhiwani	Mustard	3.217	3.221	3.238	3.178	Exponential
GRU	Hisar	Wheat	5.106	5.071	5.189	5.111	Spherical
GRU	Hisar	Mustard	4.430	4.363	4.475	4.407	Spherical
GRU	Bhiwani	Wheat	6.397	5.979	5.979	5.961	Exponential
GRU	Bhiwani	Mustard	3.601	3.367	3.387	3.343	Exponential
LSTM	Hisar	Wheat	5.071	5.054	5.151	5.080	Spherical
LSTM	Hisar	Mustard	4.325	4.257	4.391	4.365	Spherical
LSTM	Bhiwani	Wheat	6.581	6.033	6.084	6.023	Exponential
LSTM	Bhiwani	Mustard	3.923	3.339	3.390	3.373	Spherical

Results indicate that the optimal variogram model varied across crops, districts, and predictive approaches rather than a single model being universally applicable. For ML, linear variograms were most frequently selected, suggesting weak or near-linear residual spatial structure. In contrast, exponential variograms were more commonly selected for Bhiwani district across both ML and DL models, reflecting stronger short-range spatial autocorrelation under more heterogeneous agro-climatic conditions.

DL models (GRU and LSTM) predominantly favored spherical variogram models, particularly in Hisar district. This pattern suggests moderate and well-defined spatial dependence in DL residuals, consistent with the ability of RNN to capture large-scale temporal variability while retaining localized spatial structure in residual errors. Gaussian variograms were rarely selected, indicating that residual surfaces generally did not exhibit excessively smooth spatial behavior. These findings underscore the importance of adaptive, data-driven variogram selection for accurately modeling residual spatial dependence prior to kriging.

### Residual spatial patterns after kriging

**Fig 12:**
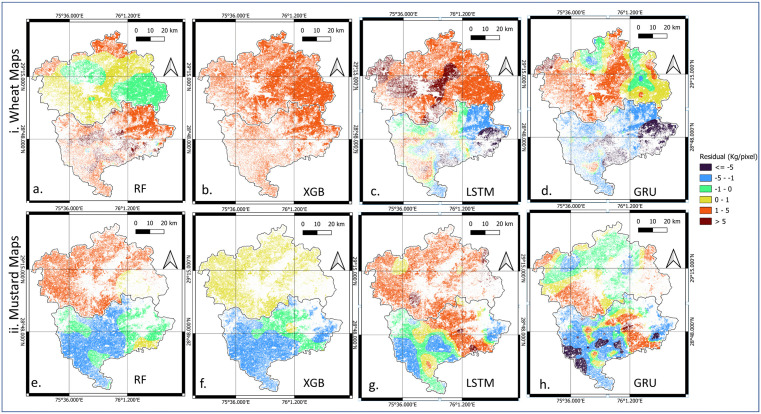
Spatial distribution of residuals after kriging for yield prediction in Hisar and Bhiwani districts: (i) wheat residual maps and (ii) mustard residual maps. These maps are analytical raster outputs (Python) produced in this study and visualized using QGIS.

The effectiveness of residual kriging was further evaluated through spatial visualization and quantitative diagnostics. Fig 12 illustrates the spatial distribution of kriged residuals for wheat and mustard yields across Hisar and Bhiwani districts. The residual maps show smoother spatial transitions and a clear reduction in localized error clusters, indicating that kriging effectively corrects spatially structured biases present in the original model outputs.

Consistent with these visual patterns, Table 8 quantitatively compares the residual *RMSE* before and after kriging and reports Moran’s I statistics. The reported p-values correspond to Moran’s I and test the null hypothesis of spatial randomness, with values below 0.05 indicating statistically significant spatial autocorrelation. Results indicate that residual spatial autocorrelation is generally weak or insignificant for ML-based models, particularly for wheat, whereas DL-based models exhibit stronger and statistically significant clustering for both wheat and mustard. Accordingly, larger reductions in residual RMSE after kriging are observed for DL-based approaches, reflecting their greater reliance on spatial correction. These findings confirm that integrating geostatistical correction with DL-based yield predictions improves both numerical accuracy and spatial realism, as evidenced by reduced residual clustering and enhanced spatial coherence in the final yield maps.

**Table 8:** Comparison of residual error, kriged residual error, and residual spatial autocorrelation across crops, districts, and predictive models.

District	Model	Crop	RMSE (kg ha ⁻ ¹)	Kriged RMSE (kg ha ⁻ ¹)	Moran’s I	p-value
Bhiwani	RF	Wheat	720.38	584.41	−0.002	0.49
Bhiwani	RF	Mustard	330.65	302.57	0.107	0.001
Bhiwani	XGB	Wheat	665.85	577.18	0.002	0.385
Bhiwani	XGB	Mustard	355.34	317.82	0.117	0.001
Bhiwani	LSTM	Wheat	691.15	602.51	0.206	0.001
Bhiwani	LSTM	Mustard	409.88	336.82	0.335	0.001
Bhiwani	GRU	Wheat	770.51	596.20	0.174	0.001
Bhiwani	GRU	Mustard	453.11	335.66	0.356	0.001
Hisar	RF	Wheat	493.39	481.97	0.059	0.017
Hisar	RF	Mustard	401.57	376.40	−0.040	0.083
Hisar	XGB	Wheat	598.42	484.66	0.003	0.377
Hisar	XGB	Mustard	392.37	389.74	−0.022	0.26
Hisar	LSTM	Wheat	668.94	505.42	0.134	0.001
Hisar	LSTM	Mustard	548.42	425.75	0.150	0.001
Hisar	GRU	Wheat	555.76	506.85	0.147	0.001
Hisar	GRU	Mustard	465.73	436.27	0.174	0.001

### Post-kriging ML and DL yield disaggregation

**Fig 13:**
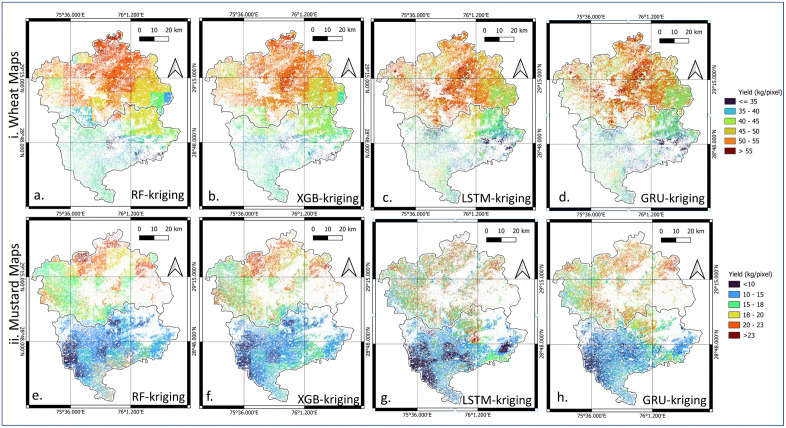
Comparison among geostatistically corrected Hisar and Bhiwani crop map of ML- kriging and DL-kriging, i. wheat map ii. mustard map. These maps are analytical raster outputs (Python) produced in this study and visualized using QGIS.

To enhance statistical accuracy and spatial realism, a hybrid method (DL-kriging) was developed. In DL methods, kriging correction effectively reduced systematic regional biases without destroying fine-scale heterogeneity (Fig 13(c-d, g-h)). In contrast, ML-kriging maps (RF-kriging, XGB-kriging) continued to exhibit exaggerated block boundaries, a residual effect of their categorical tree splits in both wheat and mustard maps (Figs 13(a-b, e-f)). Based on these visual inspection and residual behavior, the GRU-kriging approach demonstrated spatial smoothness with preservation of local variability. Consequently, GRU-kriging emerged as the most effective method for yield disaggregation using the *band-weather* dataset.

### Validation

Due to the unavailability of pixel-level GT yield data, validation was performed using officially reported village-level and block-level crop yield statistics obtained from the Department of Agriculture and Farmers Welfare, Haryana. These statistics represent aggregated average yields compiled through crop-cutting experiments and administrative reporting, and therefore differ from pixel-level GT data, which would correspond to direct yield measurements at field or pixel scale. For validation, pixel-level predictions generated from each disaggregation approaches were spatially aggregated to the respective village and block level and then compared with reported yield statistics for Bhiwani and Hisar districts (Table 9). Table 9 shows the overall performance of the different disaggregation methodologies.

**Table 9:** Village and Block level performance metrics of crop yield disaggregation models.

	Village-level					Block-level		
Model	R^2^	Ad. R^2^	RMSE	MAE	MAPE	RMSE	MAE	MAPE
Weight-Based	0.832	0.824	7.163	5.441	19.39	3.097	2.580	11.08
Percentile-Based	0.858	0.850	7.482	6.040	27.57	5.638	4.650	17.70
RF	0.916	0.911	5.336	4.029	15.28	2.447	1.919	7.65
XGB	0.909	0.904	4.930	3.666	14.59	2.830	2.314	7.57
GRU	0.876	0.870	5.667	3.873	15.74	3.067	2.385	8.88
LSTM	0.867	0.856	5.983	4.299	18.11	3.565	3.051	12.69
GRU-Kriging	0.886	0.879	5.387	3.793	15.65	1.852	1.499	6.44
LSTM-Kriging	0.884	0.878	5.425	3.806	15.43	1.965	1.512	6.5

Note: *MAE*, *RMSE* in quintal/ha; *MAPE* in % and *R^2^*. *Ad. R^2^*: *Adjusted R^2^*. *R^2^* and *adjusted R^2^* are not reported at the block level due to the limited number of block-level samples relative to the number of predictors, which can lead to unstable and non-interpretable estimates.

At the village level, performance exhibited greater variability due to limited training data and local heterogeneity in management. The traditional approaches performed reasonably (*R^2^* = 0.832–0.858) but were less reliable, due to higher *MAPE*. The ML models achieved the highest accuracy, with *R^2^* ranging from 0.909 to 0.916 and *RMSE* between 4.93–5.34 q/ha. Their ability to model nonlinear relationships between spectral and weather variables enabled stable and robust predictions even across heterogeneous agro-climatic zones. In contrast, DL models showed moderate performance (*R^2^* = 0.867–0.876; *RMSE* = 5.67–5.98 q/ha), reflecting the challenge of learning long-term temporal dependencies from limited samples. Applying residual kriging to DL outputs improved their accuracy (*R^2^* ≈ 0.885; *RMSE* ≈ 5.40 q/ha), demonstrating that even at coarse scales, this hybridization effectively mitigated systematic spatial bias.

At the block scale, where spatial aggregation reduced noise, all models achieved substantially better accuracy. The Weight-Based NDVI method produced plausible spatial patterns and reasonable accuracy (*RMSE* ≈ 3.09 q/ha; *MAPE* >11), demonstrating its utility as a simple and data-efficient baseline (Table 8). However, the Percentile-Based approach exhibited unstable behavior (*RMSE* > 5.60 q/ha, *MAPE* > 17), reflecting its sensitivity to outliers and its inability to capture temporal crop dynamics. Among all models, the ensemble ML approaches (XGB, RF) achieved the strongest numerical performance. RF (*RMSE* = 2.45 q/ha) slightly outperformed XGB (*RMSE* = 2.83 q/ha) in terms of *RMSE*. Their strength lies in capturing nonlinear interactions between spectral bands and weather variables, making them particularly effective for disaggregating village-level training data into fine-scale pixel predictions. In contrast, the DL models achieved moderate performance, with higher *RMSE* ≈ 3.06–3.57 q/ha compared to ensemble ML models. Applying residual kriging to the GRU and LSTM predictions reduced errors by approximately 35–45% while retaining the spatial continuity of the DL outputs. Specifically, for GRU the *RMSE* decreased from 3.07 to 1.85 q/ha (39.6%) and for LSTM from 3.56 to 1.96 q/ha (44.9%), confirming the effectiveness of the hybrid DL-kriging approach.

The performance of crop-specific comparison of traditional, ML, DL, and hybrid (DL-kriging) models for wheat and mustard is presented in Tables 10 and 11, respectively. For wheat (Table 10), DL-kriging hybrids clearly outperformed all others, achieving the lowest *RMSE* (≈ 1.8–1.9 q/ha) and *MAPE* (≈ 3.2–3.5%). This indicates that kriging correction effectively compensated for regional biases while retaining the fine-scale detail of DL predictions. For mustard (Table 11), absolute yields were lower and hence percentage errors were higher, but the relative pattern among models was consistent. Incorporating GRU-kriging achieved *RMSE* = 1.81 q/ha and *MAPE* = 9.43%, and similarly LSTM-kriging, achieved *RMSE* = 2.12 q/ha and *MAPE* = 9.82%.

**Table 10:** Comparative performance of block levels predicted aggregated wheat yields across algorithms in metric quintal per hectare.

Block	Actual Yield	Weight-based	Percentile-based	RF	XGB	GRU	LSTM	GRU-kriging	LSTM-kriging
Adampur	50.08	43.71	47.07	48.80	45.74	45.46	43.37	49.12	49.28
Agroha	52.41	48.94	52.70	51.22	48.35	48.00	45.76	48.72	48.96
Barwala	51.51	50.05	53.90	51.72	47.43	49.97	47.02	50.53	50.24
Hansi1	51.19	48.13	51.83	50.29	46.31	49.73	47.05	51.08	51.41
Hansi2	46.64	43.55	46.90	46.22	44.25	46.23	44.36	46.63	47.06
Hisar1	47.94	47.57	51.22	46.67	45.33	48.87	45.97	50.94	50.85
Hisar2	49.05	47.90	51.58	49.10	46.43	47.03	44.52	50.23	49.98
Narnaund	49.05	48.24	51.94	49.78	46.01	49.29	46.57	49.43	49.41
Uklana	53.71	49.62	53.44	52.49	48.80	51.03	47.66	51.82	51.02
Bawani Khera	44.60	38.62	42.95	41.73	41.43	48.37	45.88	44.30	45.30
Behal	42.69	38.99	43.36	36.92	37.66	44.96	42.35	40.88	43.58
Bhiwani	39.03	37.78	42.02	35.89	37.39	44.65	42.25	40.57	37.05
Kairu	39.86	39.45	43.88	34.92	36.82	45.19	42.80	41.12	38.42
Loharu	43.40	39.94	44.42	37.28	37.89	45.09	42.66	41.01	43.20
Siwani	42.05	37.44	41.64	37.64	38.10	43.69	40.13	39.61	40.37
Tosham	38.04	38.68	43.02	34.56	36.11	45.10	42.49	41.02	41.26
**Performance measures**	**RMSE**	**3.33**	**2.44**	**3.09**	**3.75**	**3.46**	**3.90**	**1.89**	**1.79**
	**MAPE**	**6.44**	**4.19**	**6.14**	**8.40**	**6.16**	**7.56**	**3.47**	**3.20**
	**MAE**	**2.74**	**1.95**	**2.37**	**3.57**	**2.85**	**3.38**	**1.55**	**1.44**

Note: All model Predicted Yield, Actual Yield, *MAE*, *RMSE* in quintal/ha; *MAPE* in %

**Table 11:** Comparative performance of block levels predicted aggregated mustard yields across algorithms in metric quintal per hectare.

Block	Actual Yield	Weight-based	Percentile-based	RF	XGB	GRU	LSTM	GRU-kriging	LSTM-kriging
Adampur	17.38	15.53	24.52	16.11	17.59	17.60	15.44	17.70	17.63
Agroha	21.31	17.19	27.15	18.98	20.42	19.18	16.86	18.48	18.64
Barwala	18.86	17.27	27.28	18.14	19.44	19.31	16.94	18.77	19.31
Hansi1	17.78	16.70	26.38	16.86	17.79	19.45	16.58	19.06	18.64
Hansi2	21.96	15.60	24.64	19.72	20.11	16.94	14.75	19.49	19.68
Hisar1	18.93	16.23	25.63	17.29	18.66	18.56	15.58	19.57	19.51
Hisar2	17.93	16.45	25.98	16.22	18.15	17.85	15.60	18.74	18.93
Narnaund	21.26	17.09	26.99	20.72	20.54	19.74	17.11	21.03	21.09
Uklana	21.98	17.87	28.23	20.49	20.87	21.28	17.99	21.14	20.24
Bawani Khera	11.31	13.67	22.29	13.11	13.65	16.98	15.28	14.98	17.69
Behal	12.81	13.39	21.84	13.74	14.33	15.14	13.26	13.14	11.47
Bhiwani	15.33	12.82	20.91	14.51	15.32	14.40	12.68	12.40	13.78
Kairu	14.23	13.30	21.68	16.01	15.49	15.30	13.40	13.30	13.15
Loharu	15.28	13.42	21.87	16.64	16.03	15.68	13.58	13.68	13.66
Siwani	10.05	12.14	19.80	12.38	13.41	14.73	12.19	12.72	11.33
Tosham	11.63	12.48	20.35	13.13	13.35	15.05	12.82	13.05	13.63
**Performance measures**	**RMSE**	**2.84**	**7.59**	**1.56**	**1.38**	**2.61**	**3.19**	**1.81**	**2.12**
	**MAPE**	**15.71**	**31.21**	**9.16**	**6.75**	**11.59**	**17.82**	**9.43**	**9.82**
	**MAE**	**2.38**	**3.43**	**2.47**	**2.59**	**2.38**	**2.60**	**1.48**	**1.35**

Note: All model Predicted Yield, Actual Yield, *MAE*, *RMSE* in quintal/ha; *MAPE* in %

The strong block-level performance of GRU-Kriging and LSTM-Kriging, demonstrated by low *RMSE* and *MAPE* values for both wheat and mustard, provides confidence in the accuracy of pixel-level predictions. As shown in Figs 11 and 13, based on visual comparison and statistical results (Table 9–11), indicate that the hybrid models effectively reduce systematic regional biases while retaining fine-scale spatial variability within village, resulting in smoother and more realistic yield patterns compared to non-kriged ML and DL approaches. Building on this quantitative validation, the spatial disaggregation maps highlighting the practical advantages of hybrid DL-kriging approaches in representing crop yield variability across the landscape.

Overall, The GRU-kriging combination offered the best balance by reducing artificial discontinuities, preserving local gradients. It also provides a robust combination of numerical accuracy and spatial realism, making it highly suitable for high-resolution yield disaggregation across multiple crops. This approach is particularly valuable for applications requiring smoother and spatially coherent outputs, such as precision agriculture, crop insurance, and extension services, while maintaining high predictive accuracy.

### Model generalization, limitations, and transferability

The proposed yield disaggregation framework was evaluated using a single growing season and two major crops (wheat and mustard) due to the availability of consistent village-level yield statistics and supporting datasets. While the model was not explicitly validated across multiple years or additional crop types, several aspects of the framework support its potential generalization and transferability.

First, the input features Sentinel-1 SAR, Sentinel-2 optical data, weather variables, and soil properties are globally available and crop-agnostic, enabling application across different regions and cropping systems. Second, the DL component learns generalized temporal-spectral relationships rather than crop-specific empirical thresholds, while the residual kriging step adapts locally by exploiting spatial autocorrelation in prediction errors.

Furthermore, the adaptive variogram selection employed during residual kriging allows the spatial correction process to respond to varying agro-climatic and management conditions, which is essential for model transferability across regions with differing spatial structures. While crop-specific retraining would be required to account for phenological differences, the overall workflow remains unchanged and can be readily applied to other crops and growing seasons where aggregated yield statistics are available.

Future research should explore the integration of higher-resolution and temporally dynamic weather datasets to further reduce prediction uncertainty. In the current experiments, soil properties were treated as static inputs; incorporating temporally varying soil moisture and soil sensor data may improve representation of intra-seasonal yield dynamics. Extending the framework to additional crops, agro-ecological zones, and management systems would further strengthen its generalizability and support broader operational deployment.

## Conclusion

In this study, we experimented with different spectral bands, weather data and soil data at pixel level and identified their contribution in disaggregation of macro level yield values to pixel level. This pixel level disaggregated yield data is useful to estimate household yield and avoid farmer specific response bias. Spectral bands from Sentinel-1 and Sentinel-2 along with weather features were identified as the best predictors for yield disaggregation using village yields. The experimental results in the study proved that the proposed integrated framework of geostatistical and DL methods improve the disaggregation performance comparison to non-integrated approach involving ML, DL and statistical models alone. The proposed DL-kriging framework reduced *RMSE* and *MAPE* by approximately 35–45% while generating spatially coherent yield maps that preserve within-village variability. Importantly, this framework explicitly separates the roles of data-driven learning and spatial correction: DL captures large-scale temporal and spectral relationships, while kriging exploits residual spatial autocorrelation to correct systematic regional biases. This complementary interaction makes the framework transferable and applicable to new crop or other regions where only aggregated yield statistics are available.

From a practical perspective, pixel-level yield estimates derived from the proposed framework can support household-level yield assessment while reducing farmer-specific response bias inherent in survey-based approaches. The generated fine-resolution yield maps are particularly relevant for agricultural monitoring, targeted resource allocation, and evidence-based policy planning in smallholder-dominated regions. Integration of this framework with crop insurance schemes and government monitoring platforms could further enable near-real-time yield loss assessment and transparent compensation mechanisms. To transfer the benefits of such disruptive technologies to the farmers, Government of India has initiated Digital Agriculture Mission which allows registration of farmers along with crop and land details in each season. The proposed methodology shall be useful in monitoring the farm fields and also take timely decisions regarding import/ export of a commodity based on unbiased advance estimates.

## Supporting information

S1 File Village-level observed and predicted crop yields across different modeling approaches. The file provides village-level actual and predicted crop yields (quintal/ha) across proportional, machine-learning, deep learning, and hybrid DL-kriging approaches, along with associated performance metrics (*RMSE*, *MAE*, *MAPE*, and *R^2^*).
(XLSX)

S2 File Training input features used for model development. The file contains training inputs derived from remote sensing, weather, and static variables, along with associated yield values used for model development.
(XLSX)

## References

[1] Directorate of Economics and Statistics (DES). APY Data, 2022-23. Ministry of Agriculture and Farmers Welfare, Government of India. [cited Sept 2025]. Available from: https://data.desagri.gov.in/

[2] Vogel E, Donat MG, Alexander LV, Meinshausen M, Ray DK, Karoly D, et al. The effects of climate extremes on global agricultural yields. Environ Res Lett. 2019;14(5):054010. doi: 10.1088/1748-9326/ab154b

[3] Gebbers R, Adamchuk VI. Precision agriculture and food security. Science. 2010;327(5967):828–31. doi: 10.1126/science.1183899 20150492

[4] Tilman D, Balzer C, Hill J, Befort BL. Global food demand and the sustainable intensification of agriculture. Proc Natl Acad Sci U S A. 2011;108(50):20260–4. doi: 10.1073/pnas.1116437108 22106295 PMC3250154

[5] Carter M, de Janvry A, Sadoulet E, Sarris A. Index Insurance for Developing Country Agriculture: A Reassessment. Annu Rev Resour Econ. 2017;9(1):421–38. doi: 10.1146/annurev-resource-100516-053352

[6] Press Information Bureau (PIB). Assessment of crop losses through satellite [Press release]. Ministry of Agriculture and Farmers Welfare, Government of India. 2025 Feb 11 [cited Sept 2025]. Available from: http://pib.gov.in/PressReleasePage.aspx?PRID=2101838

[7] Lobell DB, Burke MB, Tebaldi C, Mastrandrea MD, Falcon WP, Naylor RL. Prioritizing climate change adaptation needs for food security in 2030. Science. 2008;319(5863):607–10. doi: 10.1126/science.1152339 18239122

[8] Gilardelli C, Stella T, Confalonieri R, Ranghetti L, Campos-Taberner M, García-Haro FJ, et al. Downscaling rice yield simulation at sub-field scale using remotely sensed LAI data. Eur J Agron. 2019;103:108–16. doi: 10.1016/j.eja.2018.12.003

[9] Tilse MJ, Filippi P, Whelan B, Bishop TFA. Downscaling crop production data to fine scale estimates with geostatistics and remote sensing: a case study in mapping cotton fibre quality. Precision Agric. 2024;25(6):2921–57. doi: 10.1007/s11119-024-10161-w

[10] Pasquel D, Roux S, Richetti J, Cammarano D, Tisseyre B, Taylor JA. A review of methods to evaluate crop model performance at multiple and changing spatial scales. Precision Agric. 2022;23(4):1489–513. doi: 10.1007/s11119-022-09885-4

[11] Steinbuch L, Orton TG, Brus DJ. Model-Based Geostatistics from a Bayesian Perspective: Investigating Area-to-Point Kriging with Small Data Sets. Math Geosci. 2019;52(3):397–423. doi: 10.1007/s11004-019-09840-6

[12] Matheron G. Principles of geostatistics. Econ Geol. 1963;58(8):1246–66. doi: 10.2113/gsecongeo.58.8.1246

[13] Robinson TP, Metternicht G. Testing the performance of spatial interpolation techniques for mapping soil properties. Comput Electron Agric. 2006;50(2):97–108. doi: 10.1016/j.compag.2005.07.003

[14] You L, Wood S. An entropy approach to spatial disaggregation of agricultural production. Agric Syst. 2006;90(1–3):329–47. doi: 10.1016/j.agsy.2006.01.008

[15] Shirsath PB, Sehgal VK, Aggarwal PK. Downscaling Regional Crop Yields to Local Scale Using Remote Sensing. Agriculture. 2020;10(3):58. doi: 10.3390/agriculture10030058

[16] Mohanasundaram S, Kasiviswanathan KS, Purnanjali C, Santikayasa IP, Singh S. Downscaling Global Gridded Crop Yield Data Products and Crop Water Productivity Mapping Using Remote Sensing Derived Variables in the South Asia. Int J Plant Prod. 2023;17(1):1–16. doi: 10.1007/s42106-022-00223-2 36405847 PMC9648444

[17] Khan MR, de Bie CAJM, van Keulen H, Smaling EMA, Real R. Disaggregating and mapping crop statistics using hypertemporal remote sensing. Int J Appl Earth Observ Geoinformat. 2010;12(1):36–46. doi: 10.1016/j.jag.2009.09.010

[18] Baghel R, Sharma P. Historical wheat yield mapping using time-series satellite data and district-wise yield statistics over Uttar Pradesh state, India. Remote Sens Appl Soc Environ. 2022;27:100808. doi: 10.1016/j.rsase.2022.100808

[19] Folberth C, Baklanov A, Balkovič J, Skalský R, Khabarov N, Obersteiner M. Spatio-temporal downscaling of gridded crop model yield estimates based on machine learning. Agric Forest Meteorol. 2019;264:1–15. doi: 10.1016/j.agrformet.2018.09.021

[20] Chen S, Liu W, Feng P, Ye T, Ma Y, Zhang Z. Improving Spatial Disaggregation of Crop Yield by Incorporating Machine Learning with Multisource Data: A Case Study of Chinese Maize Yield. Remote Sens. 2022;14(10):2340. doi: 10.3390/rs14102340

[21] Pei J, Zou Y, Liu Y, He Y, Tan S, Wang T, et al. Downscaling Administrative-Level Crop Yield Statistics to 1 km Grids Using Multisource Remote Sensing Data and Ensemble Machine Learning. IEEE J Sel Top Appl Earth Observ Remote Sens. 2024;17:14437–53. doi: 10.1109/jstars.2024.3441252

[22] Hengl T, Nussbaum M, Wright MN, Heuvelink GBM, Gräler B. Random forest as a generic framework for predictive modeling of spatial and spatio-temporal variables. PeerJ. 2018;6:e5518. doi: 10.7717/peerj.5518 30186691 PMC6119462

[23] Talebi H, Peeters LJM, Otto A, Tolosana-Delgado R. A Truly Spatial Random Forests Algorithm for Geoscience Data Analysis and Modelling. Math Geosci. 2021;54(1):1–22. doi: 10.1007/s11004-021-09946-w

[24] Cao J, Zhang Z, Luo Y, Zhang L, Zhang J, Li Z, et al. Wheat yield predictions at a county and field scale with deep learning, machine learning, and google earth engine. Eur J Agron. 2021;123:126204. doi: 10.1016/j.eja.2020.126204

[25] Wang J, Si H, Gao Z, Shi L. Winter Wheat Yield Prediction Using an LSTM Model from MODIS LAI Products. Agriculture. 2022;12(10):1707. doi: 10.3390/agriculture12101707

[26] Wang J, Wang P, Tian H, Tansey K, Liu J, Quan W. A deep learning framework combining CNN and GRU for improving wheat yield estimates using time series remotely sensed multi-variables. Comput Electron Agric. 2023;206:107705. doi: 10.1016/j.compag.2023.107705

[27] J M, M I, N N. M-Bi-GRU-CNN: a hybrid deep learning model with optimized feature selection for enhanced crop yield prediction. Multimed Tools Appl. 2025;84(32):39787–811. doi: 10.1007/s11042-025-20747-9

[28] Murthy CS, Sesha Sai MVR, Kumari VB, Roy PS. Agricultural drought assessment at disaggregated level using AWiFS/WiFS data of Indian Remote Sensing satellites. Geocarto International. 2007;22(2):127–40. doi: 10.1080/10106040701205039

[29] Mahmood T, Löw J, Pöhlitz J, Wenzel JL, Conrad C. Estimation of 100 m root zone soil moisture by downscaling 1 km soil water index with machine learning and multiple geodata. Environ Monitor Assess. 2024;196(823). doi: 10.1007/s10661-024-12969-5 PMC11333521 39158616

[30] Kumar V, Jat HS, Sharma PC, Balwinder-Singh, Gathala MK, Malik RK, et al. Can productivity and profitability be enhanced in intensively managed cereal systems while reducing the environmental footprint of production? Assessing sustainable intensification options in the breadbasket of India. Agric Ecosyst Environ. 2018;252:132–47. doi: 10.1016/j.agee.2017.10.006 29343882 PMC5727681

[31] Gogoi L, Sarma B, Taifa P, Baruah N, Devi A, Prakash A, et al. Drought Induced Impact on Growth and Yield of Wheat and Mustard: A Comparative Study. BKAP. 2024;(Of). doi: 10.18805/bkap731

[32] Sonkar G, Mall RK, Banerjee T, Singh N, Kumar TVL, Chand R. Vulnerability of Indian wheat against rising temperature and aerosols. Environ Pollut. 2019;254(Pt A):112946. doi: 10.1016/j.envpol.2019.07.114 31376598

[33] Pillai AJ, Walia P. Heat Stress in Indian Mustard (Brassica juncea L.): A Critical Review of Impacts and Adaptation Strategies. PCBMB. 2024;25(5–6):1–11. doi: 10.56557/pcbmb/2024/v25i5-68673

[34] Mao M, Zhao H, Tang G, Ren J. In-Season Crop Type Detection by Combing Sentinel-1A and Sentinel-2 Imagery Based on the CNN Model. Agronomy. 2023;13(7):1723. doi: 10.3390/agronomy13071723

[35] Survey of India (SOI). Village level digital boundary data (Product Code: OVSF/-/10) shape files. 2024 [cited 2024 Jan]. Available from: https://onlinemaps.surveyofindia.gov.in/Digital_Product_Show.aspx

[36] Kumar V, Bansal AK. The spatial distribution of various groundwater quality parameters and perform hydro chemical studies to understand the groundwater quality status in the Bhiwani and Hisar district area. Int J Adv Acad Stud. 2025;7(6):96–9. doi: 10.33545/27068919.2025.v7.i6b.1519

[37] Kamboj M, Dahiya P, Yadav P, Mishra EP, Singh R. Prediction and projections of temperature in western Haryana through ARIMA model. Environ Ecol. 2023;41(2B):1162–70.

[38] Shaloo S, Singh RP, Jain R, Bisht H. Assessment of spatial variability of soil properties in Haryana using GIS. Ann Agri-Bio Res. 2021;26(2):169–72.

[39] Kasana A, Singh O. Groundwater Irrigation Economy of Haryana:A Glimpse into Spread, Extent and Issues. JRD. 2017;36(4):531. doi: 10.25175/jrd/2017/v36/i4/120624

[40] Muñoz-Sabater J. ERA5-Land monthly averaged data from 1981 to present. Copernicus Climate Change Service (C3S) Climate Data Store (CDS). 2019. 10.24381/cds.68d2bb30

[41] Poggio L, de Sousa LM, Batjes NH, Heuvelink GBM, Kempen B, Ribeiro E, et al. SoilGrids 2.0: producing soil information for the globe with quantified spatial uncertainty. Soil. 2021;7(1):217–40. doi: 10.5194/soil-7-217-2021

[42] Vijayakumar S, Saravanakumar R, Arulanandam M, Ilakkiya S. Google Earth Engine: empowering developing countries with large-scale geospatial data analysis—a comprehensive review. Arab J Geosci. 2024;17(4). doi: 10.1007/s12517-024-11948-x

[43] Gorelick N, Hancher M, Dixon M, Ilyushchenko S, Thau D, Moore R. Google Earth Engine: Planetary-scale geospatial analysis for everyone. Remote Sens Environ. 2017;202:18–27. doi: 10.1016/j.rse.2017.06.031

[44] Hernandez J, Lobos G, Matus I, Del Pozo A, Silva P, Galleguillos M. Using Ridge Regression Models to Estimate Grain Yield from Field Spectral Data in Bread Wheat (Triticum Aestivum L.) Grown under Three Water Regimes. Remote Sens. 2015;7(2):2109–26. doi: 10.3390/rs70202109

[45] Didari S, Talebnejad R, Bahrami M, Mahmoudi MR. Dryland farming wheat yield prediction using the Lasso regression model and meteorological variables in dry and semi-dry region. Stoch Environ Res Risk Assess. 2023;37(10):3967–85. doi: 10.1007/s00477-023-02490-5

[46] Arumugam P, Chemura A, Schauberger B, Gornott C. Remote Sensing Based Yield Estimation of Rice (Oryza Sativa L.) Using Gradient Boosted Regression in India. Remote Sens. 2021;13(12):2379. doi: 10.3390/rs13122379

[47] Draper NR, Smith H. Applied Regression Analysis. 3rd edition. New York: Wiley. 1998. doi: 10.1002/9781118625590

[48] Hoerl AE, Kennard RW. Ridge Regression: Biased Estimation for Nonorthogonal Problems. Technometrics. 1970;12(1):55–67. doi: 10.1080/00401706.1970.10488634

[49] Tibshirani R. Regression Shrinkage and Selection Via the Lasso. J R Stat Soc Ser B Methodol. 1996;58(1):267–88. doi: 10.1111/j.2517-6161.1996.tb02080.x

[50] Breiman L. Random Forests. Mach Learn. 2001;45(1):5–32. doi: 10.1023/a:1010933404324

[51] Friedman JH. Greedy function approximation: A gradient boosting machine. Ann Statist. 2001;29(5). doi: 10.1214/aos/1013203451

[52] Chen T, Guestrin C. XGBoost. In: Proceedings of the 22nd ACM SIGKDD International Conference on Knowledge Discovery and Data Mining. 2016. p. 785–94. doi: 10.1145/2939672.2939785

[53] Hochreiter S, Schmidhuber J. Long short-term memory. Neural Comput. 1997;9(8):1735–80. doi: 10.1162/neco.1997.9.8.1735 9377276

[54] Cho K, van Merrienboer B, Gulcehre C, Bahdanau D, Bougares F, Schwenk H, et al. Learning Phrase Representations using RNN Encoder–Decoder for Statistical Machine Translation. In: Proceedings of the 2014 Conference on Empirical Methods in Natural Language Processing (EMNLP). 2014. p. 1724–34. doi: 10.3115/v1/d14-1179

[55] Zheng H, Yang Z, Liu W, Liang J, Li Y. Improving deep neural networks using softplus units. In: 2015 International Joint Conference on Neural Networks (IJCNN). 2015. p. 1–4. doi: 10.1109/ijcnn.2015.7280459

[56] Willmott C, Matsuura K. Advantages of the mean absolute error (MAE) over the root mean square error (RMSE) in assessing average model performance. Clim Res. 2005;30:79–82. doi: 10.3354/cr030079

[57] Wackerly DD, Mendenhall W, Scheaffer RL. Mathematical Statistics with Applications. 7th edition. Belmont, CA: Thomson Brooks/Cole; 2008.

[58] Hengl T, Heuvelink GBM, Stein A. A generic framework for spatial prediction of soil variables based on regression-kriging. Geoderma. 2004;120(1–2):75–93. doi: 10.1016/j.geoderma.2003.08.018

[59] Journel AG, Huijbregts CJ. Mining geostatistics. London: Academic Press; 1978.

[60] Nagelkerke NJD. A note on a general definition of the coefficient of determination. Biometrika. 1991;78(3):691–2. doi: 10.1093/biomet/78.3.691

[61] Yin P, Fan X. EstimatingR^2^Shrinkage in Multiple Regression: A Comparison of Different Analytical Methods. J Exp Educ. 2001;69(2):203–24. doi: 10.1080/00220970109600656

[62] Friedman M. The Use of Ranks to Avoid the Assumption of Normality Implicit in the Analysis of Variance. J Am Stat Assoc. 1937;32(200):675–701. doi: 10.1080/01621459.1937.10503522

[63] Nemenyi P. Distribution-free multiple comparisons. Princeton: Princeton University; 1963.

[64] Chen Y. Spatial autocorrelation equation based on Moran’s index. Sci Rep. 2023;13(1):19296. doi: 10.1038/s41598-023-45947-x 37935705 PMC10630413

[65] Forkel M, Carvalhais N, Verbesselt J, Mahecha M, Neigh C, Reichstein M. Trend Change Detection in NDVI Time Series: Effects of Inter-Annual Variability and Methodology. Remote Sens. 2013;5(5):2113–44. doi: 10.3390/rs5052113

